# Reproductive characteristics, menopausal status, race and ethnicity, and risk of breast cancer subtypes defined by ER, PR and HER2 status: the Breast Cancer Etiology in Minorities study

**DOI:** 10.1186/s13058-024-01834-5

**Published:** 2024-05-31

**Authors:** Esther M. John, Jocelyn Koo, Amanda I. Phipps, Teri A. Longacre, Allison W. Kurian, Sue A. Ingles, Anna H. Wu, Lisa M. Hines

**Affiliations:** 1 Department of Epidemiology & Population Health, Stanford University School of Medicine, Stanford, CA 94305 USA; 2 Department of Medicine, Division of Oncology, Stanford University School of Medicine, Stanford, CA 94304 USA; 3 Stanford Cancer Institute, Stanford University School of Medicine, Stanford, CA 94304 USA; 4 Department of Epidemiology, University of Washington, Seattle, WA 98195 USA; 5 Epidemiology Program, Public Health Sciences Division, Fred Hutchinson Cancer Center, Seattle, WA 98109 USA; 6 Department of Pathology, Stanford University School of Medicine, Stanford, CA 94305 USA; 7 Department of Population and Public Health Sciences, Keck School of Medicine of USC, Norris Comprehensive Cancer Center, University of Southern California, Los Angeles, CA 90089 USA; 8 Department of Biology, University of Colorado at Colorado Springs, Colorado Springs, CO 80918 USA; 9 Stanford University School of Medicine, 3145 Porter Drive, Suite E223, Palo Alto, CA 94504 USA

**Keywords:** Breast cancer subtypes, Reproductive factors, Race and ethnicity, Menopausal status

## Abstract

**Background:**

Associations between reproductive factors and risk of breast cancer differ by subtype defined by joint estrogen receptor (ER), progesterone receptor (PR), and HER2 expression status. Racial and ethnic differences in the incidence of breast cancer subtypes suggest etiologic heterogeneity, yet data are limited because most studies have included non-Hispanic White women only.

**Methods:**

We analyzed harmonized data for 2,794 breast cancer cases and 4,579 controls, of whom 90% self-identified as African American, Asian American or Hispanic. Questionnaire data were pooled from three population-based studies conducted in California and data on tumor characteristics were obtained from the California Cancer Registry. The study sample included 1,530 luminal A (ER-positive and/or PR-positive, HER2-negative), 442 luminal B (ER-positive and/or PR-positive, HER2-positive), 578 triple-negative (TN; ER-negative, PR-negative, HER2-negative), and 244 HER2-enriched (ER-negative, PR-negative, HER2-positive) cases. We used multivariable unconditional logistic regression models to estimate subtype-specific ORs and 95% confidence intervals associated with parity, breast-feeding, and other reproductive characteristics by menopausal status and race and ethnicity.

**Results:**

Subtype-specific associations with reproductive factors revealed some notable differences by menopausal status and race and ethnicity. Specifically, higher parity without breast-feeding was associated with higher risk of luminal A and TN subtypes among premenopausal African American women. In contrast, among Asian American and Hispanic women, regardless of menopausal status, higher parity with a breast-feeding history was associated with lower risk of luminal A subtype. Among premenopausal women only, luminal A subtype was associated with older age at first full-term pregnancy (FTP), longer interval between menarche and first FTP, and shorter interval since last FTP, with similar OR estimates across the three racial and ethnic groups.

**Conclusions:**

Subtype-specific associations with reproductive factors overall and by menopausal status, and race and ethnicity, showed some differences, underscoring that understanding etiologic heterogeneity in racially and ethnically diverse study samples is essential. Breast-feeding is likely the only reproductive factor that is potentially modifiable. Targeted efforts to promote and facilitate breast-feeding could help mitigate the adverse effects of higher parity among premenopausal African American women.

**Supplementary Information:**

The online version contains supplementary material available at 10.1186/s13058-024-01834-5.

## Introduction

Racial and ethnic differences in the incidence of breast cancer subtypes are well documented in the Surveillance, Epidemiology, and End Results (SEER) Program [1]. Among incident cases with known subtype defined by estrogen receptor (ER), progesterone receptor (PR) and human epidermal growth factor receptor 2 (HER2) [1], luminal A (ER-positive and/or PR-positive and HER2-negative) is the most common subtype, accounting for 72.7% of breast cancers, with the highest incidence among non-Hispanic White (NHW) women. Triple negative (TN) subtype (ER-negative and PR-negative and HER2-negative) accounts for 12.2% of breast cancers, and, among women diagnosed under age 50 years, the incidence is highest among African American and Hispanic women. Luminal B (ER-positive and/or PR-positive and HER2-positive) and HER2-enriched (ER-negative and PR-negative and HER2-positive) subtypes account for 4.6% and 10.3% of breast cancers, respectively. Racial and ethnic differences in the incidence of breast cancer subtypes suggest etiologic heterogeneity. Most epidemiologic studies, however, included NHW women only [2–7]. There is a need to better understand risk factors for breast cancer subtypes among racially and ethnically minoritized populations who have a greater burden of the clinically more aggressive subtypes that have poorer prognosis compared to luminal A subtype [8].

We investigated subtype-specific associations with reproductive characteristics which are well established risk factors for breast cancer [9, 10]. Heterogeneity by subtypes has been reported, although results are not consistent [2–7]. Furthermore, most findings on subtype-specific associations with reproductive factors are based on cohort and case-control studies [11–19] and pooled analyses [4, 6, 20, 21] that included mostly NHW women; few studies have been conducted among African American women [21–25], and subtype-specific analyses among Asian American or Hispanic women are lacking. We previously examined associations between reproductive factors and risk of breast cancer defined by joint ER/PR status in the Breast Cancer Etiology in Minorities (BEM) Study, a population-based pooled dataset with 90% of study participants who self-identified as African American, Asian American, or Hispanic [26, 27]. Building upon this previous work, the present analysis was based on a subset of women with breast cancer who had complete data on ER/PR/HER2 status. There is some evidence that age at diagnosis or menopausal status may modify some subtype-specific associations with reproductive factors, but findings are not consistent [6, 7, 17, 27–32]. Given that younger women are more likely to be diagnosed with more aggressive breast cancer subtypes compared with older women [1], an evaluation of menopause-specific associations with reproductive factors is warranted. To fill these gaps in knowledge, we conducted subtype-specific case-control analyses overall and by menopausal status and race and ethnicity.

## Materials and methods

### Study sample

The analysis was based on harmonized data from three population-based studies included in the BEM Study [26]: the Los Angeles County Asian American Breast Cancer Study (AABCS), a case-control study of Chinese, Japanese, and Filipina women [33]; the San Francisco Bay Area Breast Cancer Study (SFBCS), a case-control study of Hispanic, African American, and NHW women [34]; and the Northern California Breast Cancer Family Registry (NC-BCFR), a multiethnic family study that oversampled African American, Chinese, Filipina, Japanese, and Hispanic women and also included population controls [35] (Additional file 1: Table S1). Briefly, the three studies ascertained incident female breast cancer cases through regional population-based cancer registries that are part of the California Cancer Registry and the SEER Program. In AABCS, Chinese, Japanese, and Filipina cases aged 25–74 years, diagnosed with invasive breast cancer from 1995 to 2001 or 2003 to 2006, were ascertained through the Los Angeles County Cancer Surveillance Program. In SFBCS, African American, Hispanic and NHW women diagnosed with invasive breast cancer at age 35–79 years from 1995 to 1999 (all African American women and a 10% random sample of NHW women) or 1995 to 2002 (all Hispanic women) were ascertained through the Greater Bay Area Cancer Registry. In NC-BCFR, women diagnosed with invasive breast cancer at age 18–64 years were ascertained through the Greater Bay Area Cancer Registry (diagnoses 1995 to 2009) or the Sacramento and Sierra Cancer Registry (diagnoses 2005 to 2006). Details on the eligibility criteria and sampling in NC-BCFR are provided in Additional file 1: Table S1. Population controls were identified through random digit-dialing in SFBCS and NC-BCFR or neighborhood block-walking in AABCS, and frequency-matched to cases on race and ethnicity and age group. The Institutional Review Boards of the participating institutions approved the studies, and study participants provided signed informed consent.

The present analysis included women with a first primary invasive breast cancer defined by joint ER/PR/HER2 status obtained from the regional cancer registries at each study site. Reporting of HER2 expression was not required before 1999 in California. Thus, HER2 data were available for only a subset of cases diagnosed during the early years of case ascertainment in the three studies. For 108 NC-BCFR cases diagnosed from 1995 to 1998 with data on ER/PR status, stored tumor slides were used to determine HER2 expression status by immunohistochemistry (by T.L.). Of 5,243 available controls, 20% were NHW, compared to 10% NHW cases. To achieve a more balanced pooled dataset for NHW women, we selected a random sample of available NHW controls frequency-matched to NHW cases at a 1:1.5 case-control ratio by 1-year age group. The current study sample comprised 2,840 cases and 4,653 controls, of whom 90% self-identified as non-Hispanic African American, non-Hispanic Asian American, or Hispanic (White or Black).

### Data collection and harmonization

The three studies collected information on breast cancer risk factors using structured questionnaires that were administered in English, Spanish, Cantonese or Mandarin by trained staff in home visits. Risk factors were assessed up to the reference year which was defined as the calendar year before diagnosis for cases or before the interview for controls in AABCS and NC-BCFR or the calendar year before selection into the study for controls in SFBCS. Height and weight during the reference year were assessed by self-report in the three studies, and height and weight were measured at the interview in AABCS and SFBCS.

Questionnaire data were harmonized according to common definitions [26]. Race and ethnicity were based on self-report and categorized as non-Hispanic African American, non-Hispanic Asian American, Hispanic (White or Black), or NHW. Parity was defined as the number of full-term pregnancies (FTP). Lifetime duration of breast-feeding was calculated by summing duration of breast-feeding reported as a continuous measure for each live birth, except for NC-BCFR. In that study, breast-feeding was assessed as a categorical measure (0, < 1, 1–5, 6–11, 12–24, ≥ 25 months) for each pregnancy, and the midpoint of the reported category was used, or 0.5 and 30 months for the categories  < 1 month and ≥ 25 months, respectively, to calculate lifetime duration of breast-feeding. To assess the joint association of breast-feeding and parity, we generated a composite variable (1–2 FTP/never breast-fed; 1–2 FTP/ever breast-fed; ≥3 FTP/never breast-fed; ≥3 FTP/ever breast-fed) that we and others have used previously [18, 27, 36–38]. Given that the lower breast cancer risk associated with higher parity is apparent only about 10 years after the last FTP [6], we also used a composite variable to assess the impact of time since last FTP on parity (< 10 years/1–2 FTP; <10 years/≥3 FTP; ≥10 years/1–2 FTP; ≥10 years/≥3 FTP). Women who still had menstrual periods or were pregnant, breast-feeding or perimenopausal during the reference year, and under age 55 years were classified as premenopausal. Women who reported that their periods had stopped naturally or due to surgery, medical treatment, or other reasons prior to the reference year were classified as postmenopausal. Women who still had periods when they started using menopausal hormone therapy were classified as postmenopausal if they were ≥ 55 years of age; otherwise, their menopausal status was classified as unknown. Body mass index (BMI) was calculated as self-reported weight (kg) in the reference year divided by measured or self-reported height (m) squared. If self-reported weight in the reference year was missing, measured weight was used. If measured height was missing, self-reported height was used.

### Statistical analyses

We used unconditional logistic regression models to calculate odds ratios (OR) as estimates of relative risks, in accordance with the rare disease assumption, particularly for breast cancer subtypes. We calculated OR and 95% confidence intervals (CI) for associations of breast cancer subtypes with parity, lifetime duration of breast-feeding, a composite parity/breast-feeding variable, age at menarche, age at first FTP, interval between age at menarche and first FTP, interval between last FTP and diagnosis, and a composite variable of interval between last FTP and diagnosis/parity. Because of smaller sample sizes, analyses for luminal B, TN, and HER2-enriched subtypes were based on broader exposure categories. Regression models were adjusted for race and ethnicity, study, age, education, first-degree family history of breast cancer, personal history of benign breast disease, history of oral contraceptive use, BMI in the reference year, and alcohol consumption in the reference year. Categories of the covariates are shown in the footnotes of the tables. Because the association between BMI and breast cancer risk differs by menopausal status [39], regression models for all women combined were additionally adjusted for a composite variable of menopausal status/BMI (premenopausal BMI < 25 kg/m^2^, premenopausal BMI 25-29.9, premenopausal BMI ≥ 30, postmenopausal BMI < 25, postmenopausal BMI 25-29.9, postmenopausal BMI ≥ 30, unknown menopausal status).

Among premenopausal women, we also adjusted the parity analyses for interval between last FTP and diagnosis. The OR estimates changed very minimally (results not shown) and we did not adjust for years since last FTP in the multivariable models presented in the tables. Linear trends were assessed across ordinal values of categorical variables. Separate analyses were performed for premenopausal and postmenopausal women. For comparison of findings with other studies, most of which did not stratify the analyses by menopausal status or age, we also performed analyses for all women combined that included those with unknown menopausal status. To assess heterogeneity in associations by subtype, we used polytomous regression models, and tested for differences in subtype-specific ORs using a Wald statistic p value. We tested for heterogeneity by menopausal status by including interaction terms for reproductive factors and menopausal status in unconditional logistic regression models, excluding women with unknown menopausal status. To test for heterogeneity by race and ethnicity, we included an interaction term of each exposure variable with race and ethnicity, and tested for heterogeneity using a Wald statistic p value. Among all women combined, we evaluated between-study heterogeneity in subtype-specific associations, separately for premenopausal and postmenopausal women, by including interaction terms for reproductive factors and study. We excluded 46 cases and 74 controls with missing covariate data, leaving 2,794 cases and 4,579 controls in the analytic dataset. NHW cases were only included in the TN analyses as there were only a small number of NHW cases with information on all three markers (84 luminal A, 14 luminal B, 10 HER2-enriched cases). However, because NC-BCFR recruited all TN cases diagnosed from 2007 to 2009 (see Additional file 1: Table S1), the TN case group included 165 NHW cases and analyses were stratified by the four racial and ethnic groups. Counts of controls and cases by subtype, menopausal status, race and ethnicity, and parity status are shown in Additional file 2: Table S2. Two-sided p values were used for tests of trend, with a *p* < 0.05 considered statistically significant. Statistical analyses were conducted using SAS version 9.4 software (SAS Institute, Inc., Cary, NC).

## Results

Of 2,794 breast cancer cases in the analysis, 17% self-identified as African American, 39% Asian American, 34% Hispanic, and 10% NHW (Table 1). Hispanic cases were mostly White; only 17 Hispanic cases self-identified as Black. Compared to controls, higher proportions of cases had a higher education, a first-degree family history of breast cancer, nulliparity or low parity, older age at first FTP, no breast-feeding or for ≤ 12 months, premenopausal status, and higher alcohol consumption. Distributions of reproductive factors among controls varied widely by race and ethnicity (all *p* < 0.05) (Additional file 3: Table S3). Among premenopausal controls, proportions ranged from 6 to 30% for ≥ 4 FTP, 6 to 26% for breast-feeding ≥ 24 months, 4 to 34% for first FTP at age < 20 years; and 20 to 55% for ≥ 15-year interval between menarche and first FTP.

**Table 1:** Characteristics of controls and breast cancer cases by molecular subtype

	Controls		All cases		Luminal A ^a^		Luminal B ^b^		Triple- negative ^c^		HER2-enriched ^d^	
	*N* = 4,579		*N* = 2,794		*N* = 1,530		*N* = 442		*N* = 578		*N* = 244	
	N	%	N	%	N	%	N	%	N	%	N	%
**Study**												
AABCS	1,880	41	728	26	444	29	150	34	64	11	70	29
NC-BCFR	436	10	1,652	59	837	55	222	50	451	78	142	58
SFBCS	2,263	49	414	15	249	16	70	16	63	11	32	13
**Time period** ^**e**^												
1995–1999	2,506	55	490	18	275	18	78	18	91	16	46	19
2000–2004	1,747	38	1,257	45	744	49	219	50	182	31	112	46
2005–2009	326	7	1,047	37	511	33	145	33	305	53	86	35
**Race and ethnicity**												
African American	663	14	474	17	245	16	72	16	115	20	42	17
Asian American	1,968	43	1,106	39	653	43	208	47	134	23	111	45
Hispanic ^f^	1,502	33	941	34	548	36	148	33	164	28	81	33
Non-Hispanic White	446	10	273	10	84	5	14	3	165	29	10	4
**Age (years)** ^**g**^												
<45	1,201	26	767	27	387	25	135	31	171	30	74	30
45–54	1,526	33	1,026	37	575	38	159	36	204	35	88	36
55–64	1,136	25	798	29	432	28	113	26	187	32	66	27
65–79	716	16	203	7	136	9	35	8	16	3	16	7
**Education** ^**h**^												
High school graduate or less	1,789	39	853	31	470	31	147	33	151	26	85	35
Some college or vocational/technical school	1,124	25	815	29	435	28	107	24	199	34	74	30
College or higher degree	1,666	36	1,126	40	625	41	188	43	228	39	85	35
**Family history of breast cancer** ^**h i**^												
No	4,151	91	2,308	83	1,261	82	370	84	486	84	191	78
Yes	428	9	486	17	269	18	72	16	92	16	53	22
**Personal history of benign breast disease**												
No	3,598	79	2,169	78	1,126	74	342	77	508	88	193	79
Yes	981	21	655	23	404	26	100	23	97	17	54	22
**Parity (number of FTP)** ^**h**^												
Nulliparous	636	14	594	21	332	22	98	22	127	22	37	15
1	663	14	503	18	270	18	85	19	108	19	40	16
2	1,285	28	849	30	467	31	135	31	155	27	92	38
3	901	20	479	17	258	17	68	15	115	20	38	16
≥4	1,094	24	369	13	203	13	56	13	73	13	37	15
**Lifetime breast-feeding (months), parous women** ^**h**^												
0	1,248	32	773	35	409	34	120	35	167	37	77	37
≤12	1,468	37	882	40	500	42	126	37	167	37	89	43
>12	1,227	31	545	25	289	24	98	28	117	26	41	20
**Age at menarche (years)**												
<12	913	20	589	21	315	21	96	22	129	22	49	20
12	1,104	24	717	26	407	27	104	24	144	25	62	25
13	1,165	25	704	25	361	24	121	27	153	26	69	28
≥14	1,384	30	772	28	442	29	120	27	149	26	61	25
Missing	13	< 1	12	< 1	5	< 1	1	< 1	3	1	3	1
**Age at first FTP (years), parous women** ^**h**^												
<20	792	20	414	19	208	17	61	18	103	23	42	20
20–24	1,282	33	679	31	370	31	109	32	143	32	57	28
25–29	1,113	28	606	28	316	26	107	31	111	25	72	35
≥30	743	19	501	23	304	25	67	19	94	21	36	17
Missing	13	< 1	0	0	0	0	0	0	0	0	0	0
**Menopausal status** ^**h**^												
Premenopausal	1,929	42	1,291	46	699	46	215	49	264	46	113	46
Postmenopausal	2,438	53	1,428	51	792	52	216	49	293	51	127	52
Unknown	212	5	75	3	39	3	11	2	21	4	4	2
**Body mass index (kg/m**^**2**^) ^**j**^												
<25	2,275	50	1,393	50	767	50	226	51	266	46	134	55
25-29.9	1,243	27	746	27	416	27	119	27	149	26	62	25
≥30	1,061	23	655	23	347	23	97	22	163	28	48	20
**Alcohol consumption (drinks per week)** ^**h j**^												
0	3,130	68^k^	1,952	70	1,075	70	305	69	391	68	181	74
<6	957	21	491	18	266	17	86	19	100	17	39	16
≥6	492	11	351	13	189	12	51	12	87	15	24	10

*Abbreviations* *AABCS* Asian American Breast Cancer Study, *FTP* full-term pregnancy, *HER2* human epidermal growth factor receptor 2, *NC-BCFR* Northern California Breast Cancer Family Registry, *SFBCS* San Francisco Bay Area Breast Cancer Study

^a^ Estrogen receptor-positive and/or progesterone receptor-positive, and HER2-negative

^b^ Estrogen receptor-positive and/or progesterone receptor-positive, and HER2-positive

^c^ Estrogen receptor-negative, progesterone receptor-negative, and HER2-negative

^d^ Estrogen receptor-negative, progesterone receptor-negative, and HER2-positive

^e^ Year of diagnosis (cases) or selection/interview (controls)

^f^ Includes 17 Black Hispanic cases and 6 Black Hispanic controls

^g^ Age at diagnosis (cases) or selection/interview (controls)

^h^ Chi-square p value < 0.05 for difference between controls and cases

^i^ Among first-degree relatives

^j^ In reference year

### Associations between reproductive factors and breast cancer subtypes among all women

Among all women combined, heterogeneity in associations with parity status, parity, and age at first FTP was observed across subtypes (*p* < 0.05) (Table 2). For luminal A and luminal B subtypes, parity vs. nulliparity (OR = 0.64 and 0.68) and ≥ 4 vs. 1 FTP (OR = 0.55 and 0.46) were associated with lower risk. Longer breast-feeding (> 12 vs. 0 months) was associated with lower risk of luminal A (OR = 0.69) and HER2-enriched (OR = 0.60) subtypes. For the composite of parity/breast-feeding, lower risks were observed for women with ≥ 3 FTP and a history of breast-feeding compared to those with lower parity who never breast-fed, for all subtypes, with ORs ranging from 0.55 to 0.76 and all 95% CIs excluded the null except for TN subtype. Age at menarche was not associated with risk of any subtype. Higher risk of luminal A subtype was associated with older age at first FTP (OR per year = 1.02, p-heterogeneity by subtype = 0.02).

**Table 2:** Associations between reproductive characteristics and breast cancer subtypes among all women combined

	Controls	Luminal A ^a^		Luminal B ^b^		Triple-negative ^c^		HER2-enriched ^d^	
	N	N	OR (95% CI) ^e^	N	OR (95% CI) ^e^	N	OR (95% CI) ^e^	N	OR (95% CI) ^e^
***All women***	*4,579*	*1,530*		*442*		*578*		*244*	
***Parous women***	*3,943*	*1,198*		*344*		*451*		*207*	
**Parity status**									
Nulliparous	636	332	1.0	98	1.0	127	1.0	37	1.0
Parous	3,943	1,198	0.64 (0.53–0.77)	344	0.68 (0.51–0.90)	451	0.89 (0.67–1.19)	207	1.06 (0.70–1.59)
p-heterogeneity ^f^ by subtype = 0.04									
**Parity (number of FTP)**									
1	663	270	1.0	85	1.0	108	1.0	40	1.0
2	1,285	467	0.90 (0.73–1.12)	135	0.76 (0.55–1.06)	155	0.67 (0.48–0.94)	92	1.19 (0.77–1.82)
3	901	258	0.74 (0.58–0.95)	68	0.56 (0.38–0.83)	115	0.93 (0.64–1.35)	38	0.82 (0.49–1.37)
≥ 4	1,094	203	0.55 (0.42–0.73)	56	0.46 (0.29–0.71)	73	0.64 (0.41-1.00)	37	0.91 (0.52–1.62)
p trend			< 0.01		< 0.01		0.22		0.40
Per FTP			0.85 (0.76–0.96)		0.93 (0.79–1.09)		0.93 (0.79–1.10)		0.94 (0.76–1.17)
p-heterogeneity ^f^ by subtype = 0.04									
**Lifetime breast-feeding (months), parous women**									
0	1,248	409	1.0	120	1.0	167	1.0	77	1.0
≤ 12	1,468	500	0.97 (0.80–1.17)	126	0.85 (0.63–1.15)	167	0.82 (0.61–1.10)	89	1.00 (0.69–1.43)
> 12	1,227	289	0.69 (0.56–0.87)	98	0.98 (0.70–1.37)	117	0.73 (0.52–1.02)	41	0.60 (0.38–0.95)
p trend			< 0.01		0.84		0.06		0.04
Per 12 months			0.96 (0.89–1.03)		0.94 (0.84–1.05)		0.96 (0.85–1.08)		1.01 (0.88–1.17)
p-heterogeneity ^f^ by subtype = 0.07									
**Parity (FTP) by breast-feeding**									
1–2, never	728	263	1.0	81	1.0	106	1.0	51	1.0
1–2, ever	1,220	474	0.93 (0.75–1.15)	139	0.96 (0.69–1.34)	157	0.75 (0.53–1.06)	81	1.01 (0.66–1.53)
≥ 3, never	525	147	0.79 (0.59–1.05)	40	0.79 (0.50–1.24)	62	1.06 (0.68–1.64)	28	0.95 (0.54–1.66)
≥ 3, ever	1,470	314	0.56 (0.44–0.71)	84	0.55 (0.38–0.80)	126	0.76 (0.54–1.09)	49	0.59 (0.37–0.94)
p-heterogeneity ^f^ by subtype = 0.11									
**Age at menarche (years)**									
≥ 14	1,384	442	1.0	120	1.0	149	1.0	61	1.0
13	1,165	361	0.87 (0.72–1.06)	121	1.03 (0.76–1.39)	153	0.89 (0.66–1.20)	69	1.22 (0.83–1.80)
12	1,104	407	1.10 (0.91–1.33)	104	1.02 (0.75–1.39)	144	1.18 (0.87–1.59)	62	1.30 (0.87–1.94)
< 12	913	315	0.97 (0.79–1.19)	96	1.14 (0.83–1.57)	129	1.02 (0.74–1.40)	49	1.22 (0.80–1.87)
p trend			0.65		0.47		0.49		0.30
Per year			1.00 (0.96–1.04)		1.03 (0.96–1.10)		1.01 (0.94–1.08)		1.03 (0.95–1.12)
p-heterogeneity ^f^ by subtype = 0.64									
**Age at first FTP (years)**									
< 20	792	208	1.0	61	1.0	103	1.0	42	1.0
20–24	1,282	370	1.07 (0.83–1.38)	109	1.20 (0.81–1.77)	143	0.93 (0.65–1.33)	57	0.87 (0.54–1.42)
25–29	1,113	316	1.05 (0.79–1.40)	107	1.15 (0.75–1.76)	111	0.95 (0.63–1.43)	72	1.26 (0.75–2.13)
≥ 30	743	304	1.31 (0.96–1.78)	67	0.81 (0.50–1.32)	94	0.98 (0.62–1.55)	36	0.84 (0.46–1.55)
p trend			0.09		0.29		0.98		0.99
Per year			1.02 (1.01–1.04)		0.98 (0.96–1.01)		1.00 (0.98–1.03)		1.01 (0.97–1.04)
p-heterogeneity ^f^ by subtype = 0.02									
**Interval between menarche and first FTP (years)**									
< 10	1,567	416	1.0	118	1.0	188	1.0	71	1.0
10–14	1,175	348	1.07 (0.87–1.33)	120	1.33 (0.96–1.85)	119	1.05 (0.76–1.45)	69	1.40 (0.92–2.14)
≥ 15	1,176	431	1.24 (0.98–1.56)	106	0.85 (0.58–1.23)	141	1.04 (0.73–1.47)	64	1.19 (0.75–1.89)
p trend			0.07		0.32		0.83		0.51
Per year			1.02 (0.99–1.03)		0.99 (0.96–1.01)		1.00 (0.98–1.03)		1.01 (0.97–1.04)
p-heterogeneity ^f^ by subtype = 0.09									
**Interval between last FTP and diagnosis (years)**									
≥ 20	2,224	654	1.0	175	1.0	226	1.0	116	1.0
10–19	1,038	348	1.25 (0.97–1.60)	108	1.06 (0.73–1.54)	126	1.23 (0.84–1.80)	44	0.73 (0.44–1.20)
< 10	666	196	1.24 (0.88–1.73)	61	0.78 (0.46–1.32)	99	1.43 (0.85–2.41)	47	1.00 (0.52–1.92)
p trend			0.19		0.38		0.18		0.95
Per 1 year			1.02 (1.01–1.03)		0.99 (0.97–1.01)		1.01 (0.99–1.03)		1.01 (0.98–1.05)
p-heterogeneity ^f^ by subtype = 0.10									
**Interval between last FTP and diagnosis (years) by parity (FTP)**									
≥ 10, ≥ 3	1,711	405	1.0	106	1.0	154	1.0	58	1.0
≥ 10, 1–2	1,551	597	1.45 (1.20–1.76)	177	1.64 (1.21–2.23)	198	0.98 (0.73–1.33)	102	1.54 (1.04–2.30)
< 10, ≥ 3	270	56	1.14 (0.75–1.72)	18	0.89 (0.47–1.66)	34	1.34 (0.75–2.37)	17	2.14 (1.02–4.46)
< 10, 1–2	396	140	1.42 (1.02–1.98)	43	1.15 (0.69–1.93)	65	1.06 (0.64–1.75)	30	1.67 (0.87–3.20)
p-heterogeneity ^f^ by subtype = 0.09									

*AABCS* Asian American Breast Cancer Study, *BMI* body mass index, *FTP* full-term pregnancy, *HER2* human epidermal growth factor receptor 2, *NC-BCFR* Northern California Breast Cancer Family Registry, *SFBCS* San Francisco Bay Area Breast Cancer Study

^a^ Estrogen receptor-positive and/or progesterone receptor-positive and HER2-negative

^b^ Estrogen receptor-positive and/or progesterone receptor-positive and HER2-positive

^c^ Estrogen receptor-negative and progesterone receptor-negative and HER2-negative

^d^ Estrogen receptor-negative and progesterone receptor-negative and HER2-positive

^e^ Multivariable model was adjusted for race and ethnicity (African American, Asian American, Hispanic, non-Hispanic White); study (AABCS, NC-BCFR, SFBCS); age (continuous) at diagnosis (cases) or selection/interview (controls); education (high school graduate or less, some college or vocational/technical school, college graduate or higher degree); family history of breast cancer in first-degree relatives (no, yes); personal history of benign breast disease (no, yes); parity (nulliparous, 1, 2, 3, ≥ 4 FTP); lifetime breast-feeding (nulliparous, 0, ≤ 12, >12 months); history of oral contraceptive use (never, former, current); menopausal status and BMI composite variable (premenopausal BMI < 25, premenopausal BMI 25-29.9, premenopausal BMI ≥ 30, postmenopausal BMI < 25, postmenopausal BMI 25-29.9, postmenopausal BMI ≥ 30, unknown menopausal status); and alcohol consumption in reference year (0, < 6, ≥6 drinks/week)

^f^ P heterogeneity by subtype was calculated from polytomous logistic regression models with categorical reproductive variables, using the Wald test

In analyses stratified by menopausal status (Table 3; Additional files 7–10: Figures S1-S4), associations of parity with risk of luminal A and luminal B subtypes were consistent by menopausal status. Parity was associated with lower risk of TN subtype among postmenopausal women only. Longer breast-feeding was associated with lower risk of both premenopausal (OR = 0.64, p trend = 0.02) and postmenopausal (OR = 0.76, p trend = 0.02) luminal A subtype and lower risk of HER2-enriched subtype among postmenopausal women only (OR = 0.54, p trend = 0.05). Among premenopausal women, the composite ≥ 3 FTP/ever breast-fed (vs. 1–2 FTP/never breast-fed) was associated with lower risk of luminal A subtype only (OR = 0.66), whereas among postmenopausal women, lower risks were associated with all subtypes, with ORs ranging from 0.46 to 0.64, although of borderline statistical significance for TN subtype.

**Table 3:** Associations between reproductive characteristics and breast cancer subtypes, by menopausal status

	Controls	Luminal A ^a^		Luminal B ^b^		Triple-negative ^c^		HER2-enriched ^d^	
	N	N	OR (95% CI) ^e^	N	OR (95% CI) ^e^	N	OR (95% CI) ^e^	N	OR (95% CI) ^e^
***Premenopausal women***	*1,929*	*699*		*215*		*264*		*113*	
***Parous premenopausal women***	*1,583*	*511*		*160*		*201*		*90*	
**Parity status**									
Nulliparous	346	188	1.0	55	1.0	63	1.0	23	1.0
Parous	1,583	511	0.57 (0.44–0.73)	160	0.68 (0.46–1.02)	201	1.27 (0.83–1.94)	90	0.97 (0.56–1.69)
p-heterogeneity ^f^ by subtype = 0.04									
p-heterogeneity ^g^ by menopausal status			0.38		0.58		0.03		0.81
**Parity (number of FTP)**									
1	340	149	1.0	48	1.0	58	1.0	25	1.0
2	655	216	0.83 (0.61–1.13)	68	0.66 (0.41–1.06)	71	0.53 (0.33–0.85)	39	0.93 (0.51–1.68)
3	337	95	0.73 (0.49–1.08)	28	0.51 (0.28–0.93)	43	0.85 (0.48–1.49)	17	0.91 (0.43–1.94)
≥ 4	251	51	0.67 (0.41–1.10)	16	0.46 (0.22–0.99)	29	1.14 (0.57–2.27)	9	0.95 (0.36–2.51)
p trend			0.06		0.02		0.73		0.84
Per FTP			0.79 (0.51–1.22)		0.80 (0.48–1.32)		0.98 (0.65–1.46)		1.03 (0.53-2.00)
p-heterogeneity ^f^ by subtype = 0.53									
p-heterogeneity ^g^ by menopausal status			0.65		0.79		0.01		0.52
**Lifetime breast-feeding (months), parous women**									
0	417	141	1.0	45	1.0	57	1.0	26	1.0
≤ 12	662	245	1.06 (0.78–1.45)	64	0.90 (0.56–1.45)	85	0.91 (0.57–1.46)	44	1.11 (0.62-2.00)
> 12	504	125	0.64 (0.44–0.93)	51	1.26 (0.74–2.15)	59	0.77 (0.45–1.32)	20	0.68 (0.33–1.39)
p trend			0.02		0.39		0.34		0.31
Per 12 months			0.88 (0.74–1.06)		1.00 (0.82–1.21)		0.99 (0.81–1.20)		0.85 (0.61–1.19)
p-heterogeneity ^f^ by subtype = 0.20									
p-heterogeneity ^g^ by menopausal status			0.44		0.56		0.89		0.94
**Parity (FTP) by breast-feeding**									
1–2, never	308	107	1.0	38	1.0	41	1.0	20	1.0
1–2, ever	687	258	0.95 (0.68–1.32)	78	0.91 (0.55–1.49)	88	0.84 (0.50–1.41)	44	1.07 (0.57–2.03)
≥ 3, never	112	34	0.89 (0.50–1.61)	7	0.53 (0.20–1.39)	17	1.64 (0.73–3.68)	6	1.40 (0.48–4.11)
≥ 3, ever	476	112	0.66 (0.45–0.96)	37	0.68 (0.38–1.20)	55	1.07 (0.61–1.89)	20	0.84 (0.40–1.79)
p-heterogeneity ^f^ by subtype = 0.34									
p-heterogeneity ^g^ by menopausal status			0.76		0.37		0.20		0.62
**Age at menarche (years)**									
≥14	533	169	1.0	52	1.0	56	1.0	21	1.0
13	516	166	0.98 (0.73–1.33)	55	1.04 (0.66–1.65)	71	0.97 (0.61–1.54)	41	2.04 (1.13–3.69)
12	506	217	1.45 (1.09–1.93)	54	1.11 (0.70–1.75)	77	1.32 (0.84–2.09)	28	1.66 (0.89–3.13)
< 12	372	145	1.22 (0.88–1.68)	54	1.45 (0.90–2.32)	60	1.26 (0.77–2.06)	22	1.66 (0.84–3.27)
p trend			0.03		0.14		0.18		0.23
Per year			1.06 (1.00-1.14)		1.10 (1.00-1.22)		1.06 (0.95–1.17)		1.06 (0.93–1.21)
p-heterogeneity ^f^ by subtype = 0.24									
p-heterogeneity ^g^ by menopausal status			0.37		0.71		0.86		0.09
**Age at first FTP (years)**									
< 20	257	66	1.0	27	1.0	35	1.0	16	1.0
20–24	431	120	1.27 (0.79–2.03)	45	1.57 (0.83–2.95)	60	1.49 (0.81–2.73)	18	0.81 (0.34–1.96)
25–29	466	145	1.85 (1.13–3.05)	49	1.72 (0.86–3.41)	53	1.67 (0.86–3.26)	40	2.39 (1.01–5.70)
≥ 30	427	180	2.09 (1.24–3.52)	39	0.93 (0.44–1.94)	53	1.39 (0.68–2.86)	16	0.76 (0.29–2.03)
p trend			< 0.01		0.54		0.47		0.97
Per year			1.04 (1.01–1.06)		0.98 (0.94–1.02)		1.01 (0.97–1.05)		0.99 (0.94–1.04)
p-heterogeneity ^f^ by subtype = 0.01									
p-heterogeneity ^g^ by menopausal status			0.22		0.36		0.57		0.03
**Interval between menarche and first FTP (years)**									
< 10	496	117	1.0	48	1.0	70	1.0	26	1.0
10–14	445	149	1.94 (1.32–2.86)	54	1.77 (1.04-3.00)	57	1.46 (0.88–2.43)	32	2.16 (1.08–4.32)
≥ 15	638	243	2.41 (1.60–3.61)	58	1.02 (0.57–1.81)	74	1.20 (0.69–2.08)	31	1.38 (0.66–2.89)
p trend			< 0.01		0.83		0.54		0.57
Per year			1.04 (1.01–1.07)		1.00 (0.96–1.03)		1.01 (0.98–1.05)		1.01 (0.96–1.06)
p-heterogeneity ^f^ by subtype = 0.04									
p-heterogeneity ^g^ by menopausal status			0.01		0.76		0.25		0.35
**Interval between last FTP and diagnosis (years)**									
≥ 20	258	89	1.0	28	1.0	34	1.0	19	1.0
10–19	705	241	1.47 (1.00-2.16)	74	1.29 (0.72–2.30)	77	1.12 (0.62–2.03)	28	0.58 (0.29–1.18)
< 10	617	181	1.74 (1.08–2.81)	58	1.00 (0.48–2.09)	90	1.62 (0.78–3.35)	43	1.00 (0.41–2.42)
p trend			0.02		0.89		0.15		0.87
Per year			1.02 (0.99–1.05)		1.00 (0.96–1.04)		1.00 (0.97–1.04)		0.99 (0.94–1.04)
p-heterogeneity ^f^ by subtype = 0.12									
**Interval between last FTP and diagnosis (years) by parity (FTP)**									
≥ 10, ≥ 3	343	96	1.0	27	1.0	41	1.0	10	1.0
≥ 10, 1–2	620	234	1.21 (0.84–1.74)	75	1.50 (0.86–2.61)	70	0.65 (0.38–1.13)	37	1.50 (0.67–3.40)
< 10, ≥ 3	243	50	1.15 (0.69–1.91)	17	0.80 (0.37–1.73)	31	1.20 (0.60–2.38)	16	2.58 (0.95–7.02)
< 10, 1–2	374	131	1.54 (0.98–2.44)	41	1.18 (0.58–2.40)	59	1.06 (0.53–2.10)	27	1.98 (0.75–5.25)
p-heterogeneity ^f^ by subtype = 0.16									
***Postmenopausal women*** ^***h***^	*2,438*	*792*		*216*		*293*		*127*	
***Parous postmenopausal women***	*2,177*	*659*		*175*		*234*		*114*	
**Parity status**									
Nulliparous	261	133	1.0	41	1.0	59	1.0	13	1.0
Parous	2,177	659	0.68 (0.51–0.90)	175	0.63 (0.41–0.95)	234	0.65 (0.43–0.99)	114	1.16 (0.62–2.19)
p-heterogeneity ^f^ by subtype = 0.15									
Parity (number FTP)									
1	292	114	1.0	34	1.0	44	1.0	14	1.0
2	567	236	0.92 (0.67–1.26)	62	0.78 (0.48–1.26)	81	0.81 (0.49–1.34)	52	1.55 (0.80-3.00)
3	520	158	0.73 (0.52–1.03)	40	0.61 (0.36–1.03)	67	0.89 (0.52–1.52)	20	0.83 (0.39–1.76)
≥ 4	798	151	0.51 (0.35–0.73)	39	0.46 (0.26–0.82)	42	0.48 (0.26–0.88)	28	1.04 (0.48–2.24)
p trend			< 0.01		< 0.01		0.04		0.41
Per FTP			0.87 (0.76–0.99)		0.96 (0.80–1.15)		0.99 (0.81–1.21)		0.91 (0.70–1.17)
p-heterogeneity ^f^ by subtype = 0.11									
**Lifetime breast-feeding (months), parous women**									
0	763	258	1.0	72	1.0	102	1.0	50	1.0
≤ 12	742	240	0.88 (0.69–1.13)	58	0.80 (0.54–1.18)	76	0.84 (0.56–1.26)	43	0.85 (0.53–1.37)
> 12	672	161	0.76 (0.57–1.02)	45	0.82 (0.52–1.29)	56	0.84 (0.52–1.34)	21	0.54 (0.30–0.99)
p trend			0.07		0.32		0.41		0.05
Per 12 months			0.97 (0.89–1.05)		0.88 (0.74–1.04)		0.92 (0.78–1.08)		1.05 (0.90–1.23)
p-heterogeneity ^f^ by subtype = 0.54									
**Parity (FTP) by breast-feeding**									
1–2, never	380	148	1.0	40	1.0	60	1.0	30	1.0
1–2, ever	479	202	0.93 (0.69–1.27)	56	1.02 (0.63–1.63)	65	0.85 (0.52–1.38)	36	0.99 (0.56–1.75)
≥ 3, never	385	111	0.76 (0.54–1.08)	33	0.94 (0.55–1.61)	42	0.87 (0.51–1.50)	20	0.89 (0.46–1.72)
≥ 3, ever	933	198	0.52 (0.38–0.71)	46	0.50 (0.31–0.81)	67	0.64 (0.40–1.03)	28	0.46 (0.25–0.84)
p-heterogeneity ^f^ by subtype = 0.71									
**Age at menarche (years)**									
≥14	800	259	1.0	67	1.0	86	1.0	40	1.0
13	602	186	0.83 (0.64–1.08)	62	1.01 (0.67–1.50)	76	0.76 (0.50–1.16)	26	0.77 (0.44–1.33)
12	547	180	0.92 (0.70–1.20)	46	0.92 (0.60–1.41)	63	0.98 (0.64–1.52)	33	1.16 (0.69–1.95)
< 12	479	164	0.87 (0.66–1.15)	40	0.88 (0.56–1.37)	66	0.85 (0.55–1.33)	26	1.00 (0.57–1.75)
p trend			0.40		0.51		0.72		0.70
Per year			0.97 (0.92–1.03)		0.96 (0.88–1.05)		0.96 (0.88–1.05)		1.01 (0.90–1.13)
p-heterogeneity ^f^ by subtype = 0.63									
**Age at first FTP (years)**									
< 20	489	139	1.0	33	1.0	64	1.0	25	1.0
20–24	784	240	0.96 (0.70–1.32)	62	1.01 (0.61–1.68)	77	0.74 (0.46–1.19)	39	0.89 (0.48–1.64)
25–29	608	164	0.75 (0.52–1.08)	52	0.78 (0.44–1.39)	55	0.71 (0.41–1.23)	31	0.73 (0.36–1.47)
≥ 30	285	116	1.02 (0.67–1.55)	28	0.85 (0.43–1.66)	38	0.95 (0.49–1.82)	19	0.97 (0.43–2.21)
p trend			0.65		0.41		0.80		0.76
Per year			1.01 (0.98–1.03)		0.99 (0.95–1.03)		1.01 (0.97–1.05)		1.01 (0.97–1.06)
p-heterogeneity ^f^ by subtype = 0.81									
**Interval between menarche and first FTP (years)**									
< 10	983	291	1.0	67	1.0	110	1.0	44	1.0
10–14	684	189	0.77 (0.59–1.01)	64	1.06 (0.69–1.62)	59	0.78 (0.50–1.21)	36	1.00 (0.58–1.72)
≥ 15	489	178	0.88 (0.64–1.20)	44	0.70 (0.42–1.17)	63	1.00 (0.62–1.63)	32	1.05 (0.57–1.95)
p trend			0.36		0.17		0.94		0.88
Per year			1.00 (0.98–1.02)		0.98 (0.94–1.02)		0.99 (0.96–1.03)		1.01 (0.97–1.06)
p-heterogeneity ^f^ by subtype = 0.42									

*AABCS* Asian American Breast Cancer Study, *BMI* body mass index, *FTP* full-term pregnancy, *HER2 +* human epidermal growth factor receptor 2 positive, *HER2-* human epidermal growth factor receptor 2 negative, *NC-BCFR* Northern California Breast Cancer Family Registry, *SFBCS* San Francisco Bay Area Breast Cancer Study

^a^ Estrogen receptor-positive and/or progesterone receptor-positive and HER2-negative

^b^ Estrogen receptor-positive and/or progesterone receptor-positive and HER2-positive

^c^ Estrogen receptor-negative and progesterone receptor-negative and HER2-negative

^d^ Estrogen receptor-negative and progesterone receptor-negative and HER2-positive

^e^ Multivariable model was adjusted for race and ethnicity (African American, Asian American, Hispanic, non-Hispanic White); study (AABCS, NC-BCFR, SFBCS); age (continuous) at diagnosis (cases) or selection/interview (controls); education (high school graduate or less, some college or vocational/technical school, college graduate or higher degree); family history of breast cancer in first-degree relatives (no, yes); personal history of benign breast disease (no, yes); parity (nulliparous, 1, 2, 3, ≥ 4 FTP); lifetime breast-feeding (nulliparous, 0, ≤ 12, >12 months); history of oral contraceptive use (never, former, current); BMI (< 25, 25-29.9, ≥ 30); and alcohol consumption in reference year (0, < 6, ≥6 drinks/week)

^f^ P-heterogeneity by subtype was calculated from polytomous logistic regression models with categorical reproductive variables using the Wald test

^g^ P-heterogeneity by menopausal status was calculated using the Wald test in unconditional logistic regression models with interaction terms for categorical reproductive variables and menopausal status, including only women with known menopausal status

^h^ Multivariable model was adjusted for covariates in footnote e, with history of oral contraceptive use categorized as ever vs. never use

Associations with timing of reproductive events were limited to luminal A subtype among premenopausal women, although heterogeneity by menopausal status did not reach statistical significance. Younger age at menarche was associated with higher risk of all subtypes, with ORs per year ranging from 1.06 to 1.10, although the p trend reached statistical significance only for luminal A subtype. Two-fold elevated risks were associated with older age at first FTP (≥ 30 vs. <20 years: OR = 2.09, p-heterogeneity by subtype = 0.01), longer interval between menarche and first FTP (≥ 15 vs. <10 years: OR = 2.41, p-heterogeneity by subtype = 0.04), and shorter interval since last FTP (< 10 vs. ≥20 years: OR = 1.74).

The assessment of between-study variation in subtype-specific associations, separately for premenopausal and postmenopausal women, showed no significant heterogeneity by study.

### Associations between reproductive characteristics and breast cancer subtypes by menopausal status and race and ethnicity

#### *Luminal A subtype* (African American, Asian American, and Hispanic women)

**Fig. 1:**
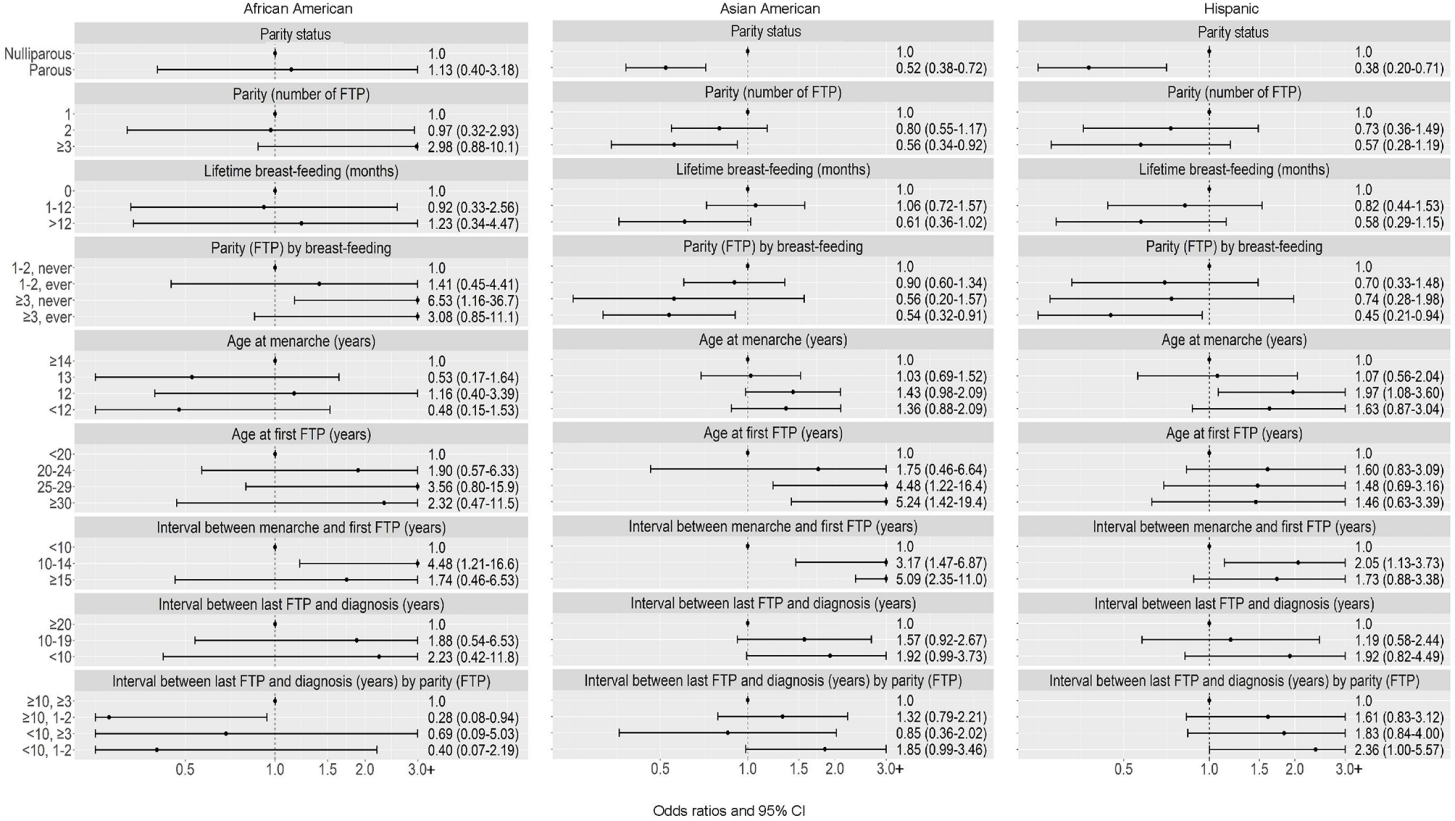
Luminal A breast cancer: Associations with reproductive characteristics among premenopausal women, by race and ethnicity

*Premenopausal women.* Associations of parity status, parity, and the composite parity/breast-feeding history with risk of luminal A subtype were generally of similar magnitude across Asian American and Hispanic participant groups (Table 4; Fig. 1). Risk of luminal A subtype was not associated with age at menarche among premenopausal African American women, whereas for Asian American and Hispanic women, OR per year were 1.10 and 1.16, respectively. Higher risks were associated with older age at first FTP, longer interval between menarche and first FTP, and shorter interval since last FTP across the three racial and ethnic groups, with estimates of OR per year generally of similar magnitude. For the composite < 10 years since last FTP/1–2 FTP (vs. ≥10 years/≥3 1FTP), suggestive higher risks were observed among Asian American (OR = 1.85, 95% CI = 0.99–3.46) and Hispanic (OR = 2.36, 95% CI = 1.00-5.57) women, with no association among African American women.

**Table 4:** Luminal A breast cancer: Associations with reproductive characteristics, by menopausal status and race and ethnicity ^a^

	All			African American			Asian American			Hispanic		
	Cs N	Cn N	OR (95% CI) ^b^	Cs N	Cn N	OR (95% CI) ^b^	Cs N	Cn N	OR (95% CI) ^b^	Cs N	Cn N	OR (95% CI) ^b^
***Premenopausal women***	*667*	*1,754*		*94*	*195*		*327*	*1,036*		*246*	*523*	
***Parous premenopausal women***	*491*	*1,474*		*76*	*164*		*223*	*828*		*192*	*482*	
**Parity status**												
Nulliparous	176	280	1.0	18	31	1.0	104	208	1.0	54	41	1.0
Parous	491	1,474	0.53 (0.40–0.69)	76	164	1.13 (0.40–3.18)	223	828	0.52 (0.38–0.72)	192	482	0.38 (0.20–0.71)
p-heterogeneity ^c^ by race and ethnicity = 0.09												
p-heterogeneity ^d^ by menopausal status			0.36			0.10			0.49			0.06
**Parity (number of FTP)**												
1	145	312	1.0	24	48	1.0	78	212	1.0	43	52	1.0
2	205	597	0.79 (0.58–1.09)	23	65	0.97 (0.32–2.93)	106	401	0.80 (0.55–1.17)	76	131	0.73 (0.36–1.49)
≥ 3	141	565	0.67 (0.46–0.97)	29	51	2.98 (0.88–10.1)	39	215	0.56 (0.34–0.92)	73	299	0.57 (0.28–1.19)
p trend			0.03			0.10			0.02			0.13
Per FTP			0.91 (0.70–1.17)			1.06 (0.51–2.22)			1.34 (0.83–2.18)			0.72 (0.50–1.04)
p-heterogeneity ^c^ by race and ethnicity = 0.07												
p-heterogeneity ^d^ by menopausal status			0.38			0.04			0.47			0.65
**Lifetime breast-feeding (months), parous women**												
0	139	398	1.0	29	86	1.0	61	214	1.0	49	98	1.0
≤ 12	234	615	1.03 (0.75–1.42)	28	52	0.92 (0.33–2.56)	126	401	1.06 (0.72–1.57)	80	162	0.82 (0.44–1.53)
> 12	118	461	0.61 (0.42–0.91)	19	26	1.23 (0.34–4.47)	36	213	0.61 (0.36–1.02)	63	222	0.58 (0.29–1.15)
p trend			0.02			0.82			0.09			0.11
Per 12 months			0.85 (0.72–1.01)			0.84 (0.48–1.44)			0.74 (0.54–0.99)			0.99 (0.78–1.24)
p-heterogeneity ^c^ by race and ethnicity = 0.26												
p-heterogeneity ^d^ by menopausal status			0.28			0.28			0.23			0.42
**Parity (FTP) by breast-feeding**												
1–2, never	106	294	1.0	20	63	1.0	56	181	1.0	30	50	1.0
1–2, ever	244	615	0.92 (0.66–1.29)	27	50	1.41 (0.45–4.41)	128	432	0.90 (0.60–1.34)	89	133	0.70 (0.33–1.48)
≥ 3, never	33	107	0.88 (0.48–1.62)	9	23	6.53 (1.16–36.7)	5	36	0.56 (0.20–1.57)	19	48	0.74 (0.28–1.98)
≥ 3, ever	108	458	0.62 (0.42–0.91)	20	28	3.08 (0.85–11.1)	34	179	0.54 (0.32–0.91)	54	251	0.45 (0.21–0.94)
p-heterogeneity ^c^ by race and ethnicity = 0.12												
p-heterogeneity ^d^ by menopausal status			0.68			0.04			0.65			0.76
**Age at menarche (years)**												
≥14	158	494	1.0	27	42	1.0	72	291	1.0	59	161	1.0
13	156	458	1.01 (0.73–1.38)	22	50	0.53 (0.17–1.64)	83	280	1.03 (0.69–1.52)	51	128	1.07 (0.56–2.04)
12	210	453	1.53 (1.13–2.07)	30	55	1.16 (0.40–3.39)	106	289	1.43 (0.98–2.09)	74	109	1.97 (1.08–3.60)
< 12	141	348	1.27 (0.91–1.78)	15	48	0.48 (0.15–1.53)	65	176	1.36 (0.88–2.09)	61	124	1.63 (0.87–3.04)
p trend			0.02			0.50			0.05			0.04
Per year			1.08 (1.01–1.16)			0.95 (0.77–1.16)			1.10 (1.01–1.21)			1.16 (1.02–1.33)
p-heterogeneity ^c^ by race and ethnicity = 0.21												
p-heterogeneity ^d^ by menopausal status			0.27			0.35			0.01			0.61
**Age at first FTP pregnancy (years)**												
< 20	64	252	1.0	23	56	1.0				38	159	1.0
20–24	112	396	1.30 (0.79–2.12)	24	60	1.90 (0.57–6.33)	25	208	1.0	66	165	1.60 (0.83–3.09)
25–29	142	433	2.12 (1.26–3.56)	14	29	3.56 (0.80–15.9)	88	314	2.80 (1.62–4.84)	40	90	1.48 (0.69–3.16)
≥ 30	173	391	2.44 (1.41–4.20)	15	19	2.32 (0.47–11.5)	110	306	3.27 (1.85–5.77)	48	66	1.46 (0.63–3.39)
p trend			< 0.01			0.18			< 0.01			0.43
Per year			1.05 (1.02–1.08)			1.09 (0.99–1.20)			1.05 (1.02–1.09)			1.02 (0.97–1.07)
p-heterogeneity ^c^ by race and ethnicity = 0.29												
p-heterogeneity ^d^ by menopausal status			0.05			0.63			< 0.01			0.37
**Interval between menarche and first FTP (years)**												
< 10	111	470	1.0	37	93	1.0	10	121	1.0	64	256	1.0
10–14	144	414	2.16 (1.43–3.25)	20	38	4.48 (1.21–16.6)	59	254	3.17 (1.47–6.87)	65	122	2.05 (1.13–3.73)
≥ 15	234	587	2.86 (1.86–4.39)	19	33	1.74 (0.46–6.53)	153	453	5.09 (2.35-11.0)	62	101	1.73 (0.88–3.38)
p trend			< 0.01			0.25			< 0.01			0.08
Per year			1.05 (1.02–1.08)			1.06 (0.97–1.16)			1.06 (1.02–1.10)			1.04 (0.99–1.09)
p-heterogeneity ^c^ by race and ethnicity = 0.17												
p-heterogeneity ^d^ by menopausal status			< 0.01			0.13			< 0.01			0.03
**Interval between last FTP and diagnosis (years)**												
≥20	86	239	1.0	22	61	1.0	30	105	1.0	34	73	1.0
10–19	231	670	1.56 (1.04–2.32)	36	69	1.88 (0.54–6.53)	116	388	1.57 (0.92–2.67)	79	213	1.19 (0.58–2.44)
< 10	174	563	1.99 (1.21–3.27)	18	34	2.23 (0.42–11.8)	77	335	1.92 (0.99–3.73)	79	194	1.92 (0.82–4.49)
p trend			< 0.01			0.34			0.06			0.10
Per year			1.03 (1.01–1.06)			1.03 (0.95–1.12)			1.03 (0.99–1.07)			1.03 (0.98–1.07)
p-heterogeneity ^c^ by race and ethnicity = 0.47												
**Interval between last FTP and diagnosis (years) by parity (FTP)**												
≥ 10, ≥ 3	93	334	1.0	23	37	1.0	29	131	1.0	41	166	1.0
≥ 10, 1–2	224	575	1.23 (0.84–1.79)	35	93	0.28 (0.08–0.94)	117	362	1.32 (0.79–2.21)	72	120	1.61 (0.83–3.12)
< 10, ≥ 3	48	229	1.20 (0.70–2.03)	6	14	0.69 (0.09–5.03)	10	84	0.85 (0.36–2.02)	32	131	1.83 (0.84-4.00)
< 10, 1–2	126	334	1.74 (1.08–2.78)	12	20	0.40 (0.07–2.19)	67	251	1.85 (0.99–3.46)	47	63	2.36 (1.00-5.57)
p-heterogeneity ^c^ by race and ethnicity = 0.11												
***Postmenopausal women*** ^***e***^	*774*	*2,201*		*150*	*430*		*313*	*904*		*281*	*867*	
***Parous postmenopausal women***	*619*	*1,979*		*116*	*381*		*246*	*775*		*257*	*823*	
**Parity status**												
Nulliparous	125	222	1.0	34	49	1.0	67	129	1.0	24	44	1.0
Parous	619	1,979	0.63 (0.47–0.86)	116	381	0.54 (0.23–1.29)	246	775	0.60 (0.41–0.87)	257	823	0.91 (0.44–1.90)
p-heterogeneity ^c^ by race and ethnicity = 0.39												
**Parity (number of FTP)**												
1	106	256	1.0	32	65	1.0	43	117	1.0	31	74	1.0
2	221	485	0.91 (0.65–1.28)	39	86	0.68 (0.25–1.82)	113	262	1.07 (0.67–1.68)	69	137	0.74 (0.39–1.42)
≥ 3	292	1,238	0.57 (0.41–0.81)	45	230	0.48 (0.18–1.24)	90	396	0.59 (0.37–0.96)	157	612	0.54 (0.29–0.99)
P trend			< 0.01			0.12			0.01			0.03
Per FTP			0.85 (0.77–0.94)			0.69 (0.43–1.10)			0.93 (0.79–1.10)			0.83 (0.73–0.94)
p-heterogeneity ^c^ by race and ethnicity = 0.90												
**Lifetime breast-feeding (months), parous women**												
0	244	679	1.0	76	200	1.0	83	225	1.0	85	254	1.0
≤ 12	222	670	0.84 (0.64–1.09)	31	104	1.05 (0.45–2.43)	106	317	0.80 (0.55–1.16)	85	249	0.90 (0.58–1.39)
> 12	153	630	0.77 (0.57–1.05)	9	77	0.50 (0.16–1.56)	57	233	0.74 (0.47–1.18)	87	320	0.94 (0.59–1.49)
p trend			0.09			0.35			0.17			0.77
Per 12 months			0.97 (0.89–1.05)			0.63 (0.33–1.21)			1.08 (0.95–1.22)			0.95 (0.86–1.05)
p-heterogeneity ^c^ by race and ethnicity = 0.90												
**Parity (FTP) by breast-feeding**												
1–2, never	140	328	1.0	44	93	1.0	56	145	1.0	40	90	1.0
1–2, ever	187	413	0.92 (0.67–1.28)	27	58	1.33 (0.48–3.69)	100	234	0.92 (0.60–1.40)	60	121	0.81 (0.43–1.52)
≥ 3, never	105	353	0.74 (0.51–1.07)	32	107	0.85 (0.33–2.17)	28	82	0.82 (0.46–1.46)	45	164	0.62 (0.34–1.15)
≥ 3, ever	187	885	0.48 (0.34–0.66)	13	123	0.39 (0.13–1.14)	62	314	0.42 (0.27–0.66)	112	448	0.56 (0.33–0.96)
p-heterogeneity ^c^ by race/ethnicity = 0.76												
**Age at menarche (years)**												
≥14	244	747	1.0	38	123	1.0	131	318	1.0	75	306	1.0
13	175	524	0.84 (0.63–1.11)	36	118	1.01 (0.43–2.36)	71	208	0.65 (0.45–0.96)	68	198	1.33 (0.82–2.16)
12	166	494	0.93 (0.70–1.23)	39	104	1.64 (0.67–3.99)	62	222	0.63 (0.43–0.93)	65	168	1.61 (0.98–2.63)
< 12	157	427	0.92 (0.68–1.24)	37	84	0.96 (0.40–2.34)	48	156	0.52 (0.34–0.81)	72	187	2.00 (1.23–3.24)
p trend			0.63			0.77			< 0.01			< 0.01
Per year			0.97 (0.92–1.03)			1.01 (0.85–1.20)			0.88 (0.81–0.95)			1.13 (1.02–1.26)
p-heterogeneity ^c^ by race and ethnicity < 0.01												
**Age at first FTP (years)**												
< 20	135	457	1.0	51	182	1.0	15	49	1.0	69	226	1.0
20–24	222	705	0.93 (0.66–1.31)	41	139	1.09 (0.46–2.58)	74	248	0.66 (0.33–1.34)	107	318	1.06 (0.68–1.67)
25–29	155	552	0.69 (0.47–1.03)	14	40	0.60 (0.18–1.99)	95	325	0.58 (0.28–1.20)	46	187	0.64 (0.36–1.14)
≥ 30	107	254	0.91 (0.58–1.42)	10	20	1.06 (0.18–6.25)	62	153	0.64 (0.29–1.39)	35	81	1.22 (0.63–2.36)
p trend			0.34			0.67			0.44			0.73
Per year			1.00 (0.98–1.03)			0.99 (0.91–1.08)			1.01 (0.97–1.05)			1.00 (0.96–1.03)
p-heterogeneity ^c^ by race and ethnicity = 0.73												
**Interval between menarche and first FTP (years)**												
< 10	275	901	1.0	80	278	1.0	59	198	1.0	136	425	1.0
10–14	177	619	0.73 (0.54–0.98)	16	69	0.67 (0.25–1.79)	87	312	0.63 (0.40–0.98)	74	238	0.88 (0.57–1.36)
≥ 15	166	439	0.81 (0.58–1.13)	20	33	0.89 (0.28–2.82)	100	265	0.77 (0.48–1.25)	46	141	0.79 (0.46–1.36)
p trend			0.19			0.66			0.48			0.37
Per year			0.99 (0.97–1.02)			0.99 (0.91–1.07)			0.98 (0.95–1.02)			1.01 (0.97–1.05)
p-heterogeneity ^c^ by race and ethnicity = 0.83												

*AABCS* Asian American Breast Cancer Study, *BMI* body mass index, *FTP* full-term pregnancy, *NC-BCFR* Northern California Breast Cancer Family Registry, *SFBCS* San Francisco Bay Area Breast Cancer Study

^a^ Associations for NHW women were not assessed since the pooled dataset included only 84 NHW women with luminal A breast cancer

^b^ Multivariable model was adjusted for study (AABCS, NC-BCFR, SFBCS); age (continuous) at diagnosis (cases) or selection/interview (controls); education (high school graduate or less, some college or vocational/technical school, college graduate or higher degree); family history of breast cancer in first-degree relatives (no, yes); personal history of benign breast disease (no, yes); parity (nulliparous, 1, 2, 3, ≥ 4 FTP); lifetime breast-feeding (nulliparous, 0, ≤ 12, >12 months); history of oral contraceptive use (never, former, current); and BMI (< 25, 25-29.9 ≥ 30); and alcohol consumption in reference year (0, < 6, ≥6 drinks/week)

^c^ P-heterogeneity by race and ethnicity using the Wald test

^d^ P-heterogeneity by menopausal status using the Wald test

^e^ Multivariable model for postmenopausal women was adjusted for covariates in footnote b, with history of oral contraceptive use categorized as ever vs. never use

**Fig. 2:**
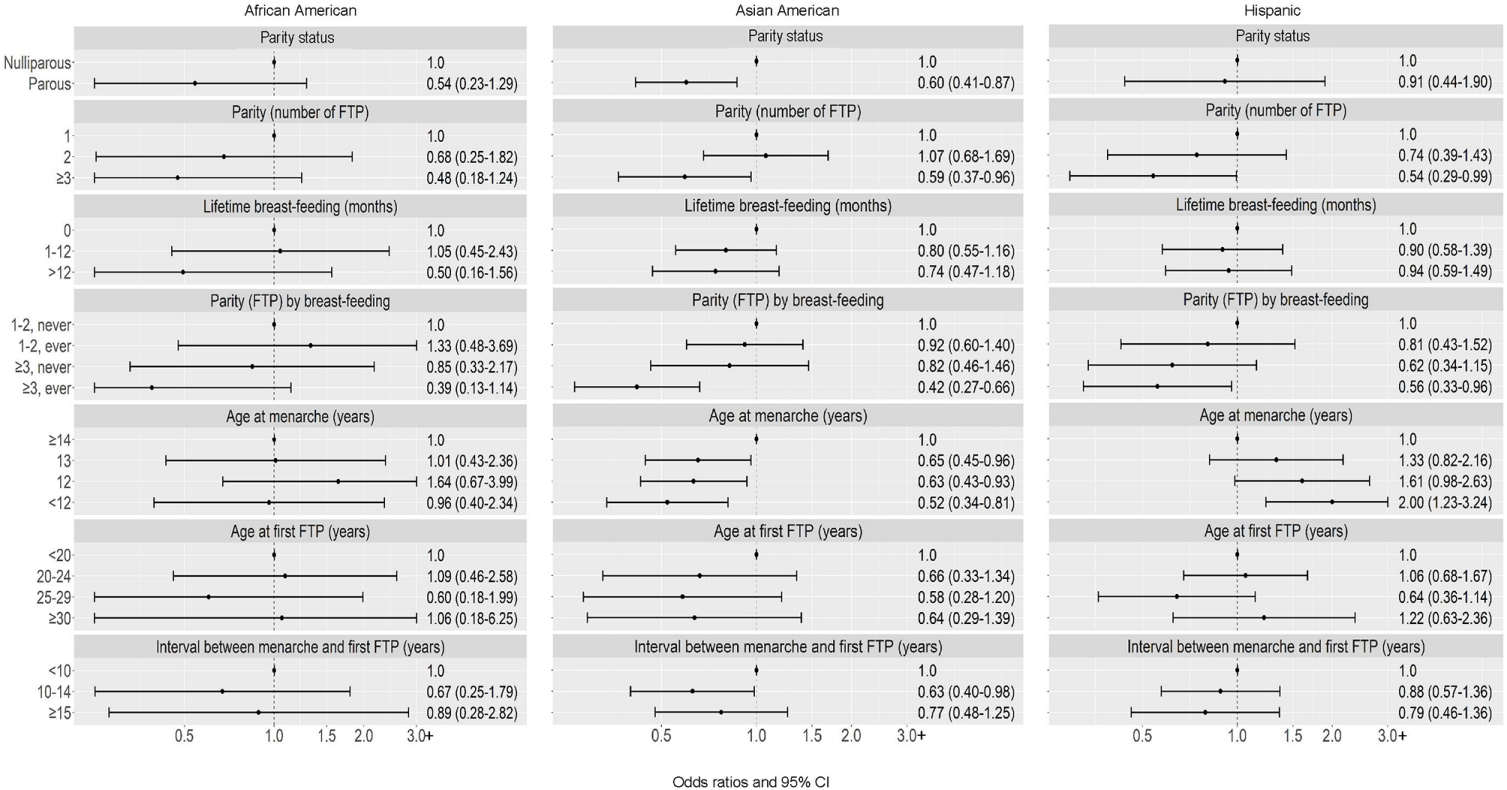
Luminal A breast cancer: Associations with reproductive characteristics among postmenopausal women, by race and ethnicity

*Postmenopausal women.* For parity status, parity, and breast-feeding, no heterogeneity by race and ethnicity was observed (Fig. 2). Higher parity (≥ 3 vs. 1 FTP) was associated with lower risk of luminal A subtype across racial and ethnic groups, with ORs ranging from 0.48 to 0.59. Lower risk was associated with the composite of higher parity with breast-feeding (vs. low parity without breast-feeding) across groups, with OR estimates ranging from 0.39 to 0.56. For age at menarche, we observed heterogeneity by race and ethnicity (*p* < 0.01). Earlier menarche (< 12 vs. ≥14 years) was associated with higher risk of luminal A subtype among postmenopausal Hispanic women only (OR = 2.00); no association was observed among African American women, whereas among Asian American women, there was an inverse association (OR = 0.52).

#### *Luminal B subtype* (African American, Asian American, and Hispanic women)

Few reproductive factors were associated with risk of luminal B subtype (Table 5). Among premenopausal women, heterogeneity by race and ethnicity was observed for parity (*p* = 0.04), breast-feeding history (*p* < 0.01), and interval between last FTP and diagnosis (*p* = 0.03). Higher parity was associated with lower risk among premenopausal Asian American (OR = 0.45) and Hispanic (OR = 0.33) women, but not among premenopausal African American women. Among postmenopausal women, higher parity (≥ 3 vs. 1–2 FTP) was associated with lower risk overall (OR = 0.57), with OR estimates of similar magnitude across the three racial and ethnic groups, ranging from 0.56 to 0.66. Lower risk was associated with older age at first FTP among Hispanic women and earlier menarche among Asian American women.

**Table 5:** Luminal B breast cancer: Associations with reproductive characteristics, by menopausal status and race and ethnicity ^a^

	All			African American			Asian American			Hispanic		
	Cs N	Cn N	OR (95% CI) ^b^	Cs N	Cn N	OR (95% CI) ^b^	Cs N	Cn N	OR (95% CI) ^b^	Cs N	Cn N	OR (95% CI) ^b^
***Premenopausal women***	*211*	*1,754*		*38*	*195*		*104*	*1,036*		*69*	*523*	
***Parous premenopausal women***	*158*	*1,474*		*28*	*164*		*71*	*828*		*59*	*482*	
**Parity status**												
Nulliparous	53	280	1.0	10	31	1.0	33	208	1.0	10	41	1.0
Parous	158	1,474	0.68 (0.45–1.02)	28	164	0.68 (0.19–2.35)	71	828	0.56 (0.34–0.91)	59	482	0.89 (0.31–2.52)
p-heterogeneity ^c^ by race and ethnicity = 0.54												
p-heterogeneity ^d^ by menopausal status			0.53			0.67			0.93			0.49
**Parity (number of FTP)**												
1–2	115	909	1.0	17	113	1.0	61	613	1.0	37	183	1.0
≥ 3	43	565	0.57 (0.36–0.93)	11	51	2.02 (0.54–7.61)	10	215	0.45 (0.22–0.94)	22	299	0.33 (0.14–0.75)
p-heterogeneity ^c^ by race and ethnicity = 0.04												
p-heterogeneity ^d^ by menopausal status			0.38			0.07			0.49			0.99
**History of breast-feeding, parous women**												
Never	45	401	1.0	7	86	1.0	26	217	1.0	12	98	1.0
Ever	113	1,073	1.01 (0.65–1.59)	21	78	3.07 (0.88–10.7)	45	611	0.61 (0.35–1.06)	47	384	2.08 (0.78–5.50)
p-heterogeneity ^c^ by race and ethnicity < 0.01												
p-heterogeneity ^d^ by menopausal status			0.77			0.16			0.16			0.11
**Age at menarche (years)**												
≥ 13	105	952	1.0	16	92	1.0	49	571	1.0	40	289	1.0
< 13	106	801	1.23 (0.88–1.72)	22	103	1.27 (0.45–3.57)	55	465	1.37 (0.89–2.10)	29	233	0.91 (0.46–1.79)
p-heterogeneity ^c^ by race and ethnicity = 0.64												
p-heterogeneity ^d^ by menopausal status			0.32			0.66			0.03			0.42
**Age at first FTP (years)**												
< 25	70	648	1.0	20	116	1.0	16	208	1.0	34	324	1.0
≥ 25	88	824	1.04 (0.65–1.68)	8	48	1.84 (0.48–7.07)	55	620	0.97 (0.50–1.87)	25	156	1.03 (0.43–2.46)
p-heterogeneity ^c^ by race and ethnicity = 0.89												
p-heterogeneity ^d^ by menopausal status			0.66			0.66			0.68			0.04
**Interval between menarche and first FTP (years)**												
< 11	60	554	1.0	17	102	1.0	14	164	1.0	29	288	1.0
≥ 11	98	917	1.01 (0.62–1.64)	11	62	1.45 (0.41–5.16)	57	664	0.85 (0.43–1.71)	30	191	1.02 (0.44–2.37)
p-heterogeneity ^c^ by race and ethnicity = 0.89												
p-heterogeneity ^d^ by menopausal status			0.67			0.63			0.65			0.32
**Interval between last FTP and diagnosis (years)**												
≥ 10	101	909	1.0	17	130	1.0	49	493	1.0	35	286	1.0
< 10	57	563	0.80 (0.48–1.34)	11	34	4.19 (0.81–21.8)	22	335	0.49 (0.24-1.00)	24	194	0.95 (0.38–2.34)
p-heterogeneity ^c^ by race and ethnicity = 0.03												
***Postmenopausal women*** ^***e***^	*207*	*2,201*		*32*	*430*		*100*	*904*		*75*	*867*	
***Parous postmenopausal women***	*168*	*1,979*		*25*	*381*		*80*	*775*		*63*	*823*	
**Parity status**												
Nulliparous	39	222	1.0	7	49	1.0	20	129	1.0	12	44	1.0
Parous	168	1,979	0.61 (0.40–0.94)	25	381	0.65 (0.21–2.03)	80	775	0.63 (0.36–1.09)	63	823	0.52 (0.21–1.26)
p-heterogeneity ^c^ by race and ethnicity = 0.99												
**Parity (number of FTP)**												
1–2	93	741	1.0	16	151	1.0	47	379	1.0	30	211	1.0
≥ 3	75	1,238	0.57 (0.38–0.84)	9	230	0.66 (0.22–1.98)	33	396	0.57 (0.33–0.99)	33	612	0.56 (0.29–1.06)
p-heterogeneity ^c^ by race and ethnicity = 0.83												
**History of breast-feeding, parous women**												
Never	70	681	1.0	17	200	1.0	27	227	1.0	26	254	1.0
Ever	98	1,298	0.79 (0.55–1.13)	8	181	0.71 (0.25–2.05)	53	548	0.94 (0.56–1.56)	37	569	0.64 (0.35–1.18)
p-heterogeneity ^c^ by race and ethnicity = 0.51												
**Age at menarche (years)**												
≥ 13	126	1,271	1.0	13	241	1.0	66	526	1.0	47	504	1.0
< 13	80	921	0.85 (0.61–1.18)	19	188	1.63 (0.67–3.95)	34	378	0.63 (0.40–0.99)	27	355	1.03 (0.58–1.84)
p-heterogeneity ^c^ by race and ethnicity = 0.06												
**Age at first FTP (years)**												
< 25	90	1,162	1.0	18	321	1.0	25	297	1.0	47	544	1.0
≥ 25	78	806	0.78 (0.52–1.17)	7	60	1.19 (0.31–4.48)	55	478	1.11 (0.63–1.96)	16	268	0.45 (0.22–0.90)
p-heterogeneity ^c^ by race and ethnicity = 0.11												
**Interval between menarche and first FTP (years)**												
< 11	79	1,022	1.0	16	294	1.0	26	246	1.0	37	482	1.0
≥ 11	89	937	0.70 (0.46–1.06)	9	86	1.08 (0.31–3.75)	54	529	0.59 (0.33–1.07)	26	322	0.71 (0.38–1.36)
p-heterogeneity ^c^ by race and ethnicity = 0.48												

*AABCS* Asian American Breast Cancer Study, *BMI* body mass index, *FTP* full-term pregnancy, *NC-BCFR* Northern California Breast Cancer Family Registry, *SFBCS* San Francisco Bay Area Breast Cancer Study

^a^ Associations for NHW women were not assessed since the pooled dataset included only 14 NHW women with luminal B breast cancer

^b^ Multivariable model was adjusted for study (AABCS, NC-BCFR, SFBCS); age (continuous) at diagnosis (cases) or selection/interview (controls); education (high school graduate or less, some college or vocational/technical school, college graduate or higher degree); family history of breast cancer in first-degree relatives (no, yes); personal history of benign breast disease (no, yes); parity (nulliparous, 1, 2, 3, ≥ 4 FTP); lifetime breast-feeding (nulliparous, 0, ≤ 12, >12 months); history of oral contraceptive use (never, former, current); and BMI (< 25, 25-29.9 ≥ 30); and alcohol consumption in reference year (0, < 6, ≥6 drinks/week)

^c^ P-heterogeneity by race and ethnicity using the Wald test

^d^ P-heterogeneity by menopausal status using the Wald test

^e^ Multivariable model for postmenopausal women was adjusted for covariates in footnote b, with history of oral contraceptive use categorized as ever vs. never use

#### *Triple-negative subtype* (African American, Asian American, Hispanic women, and NHW women)

**Fig. 3:**
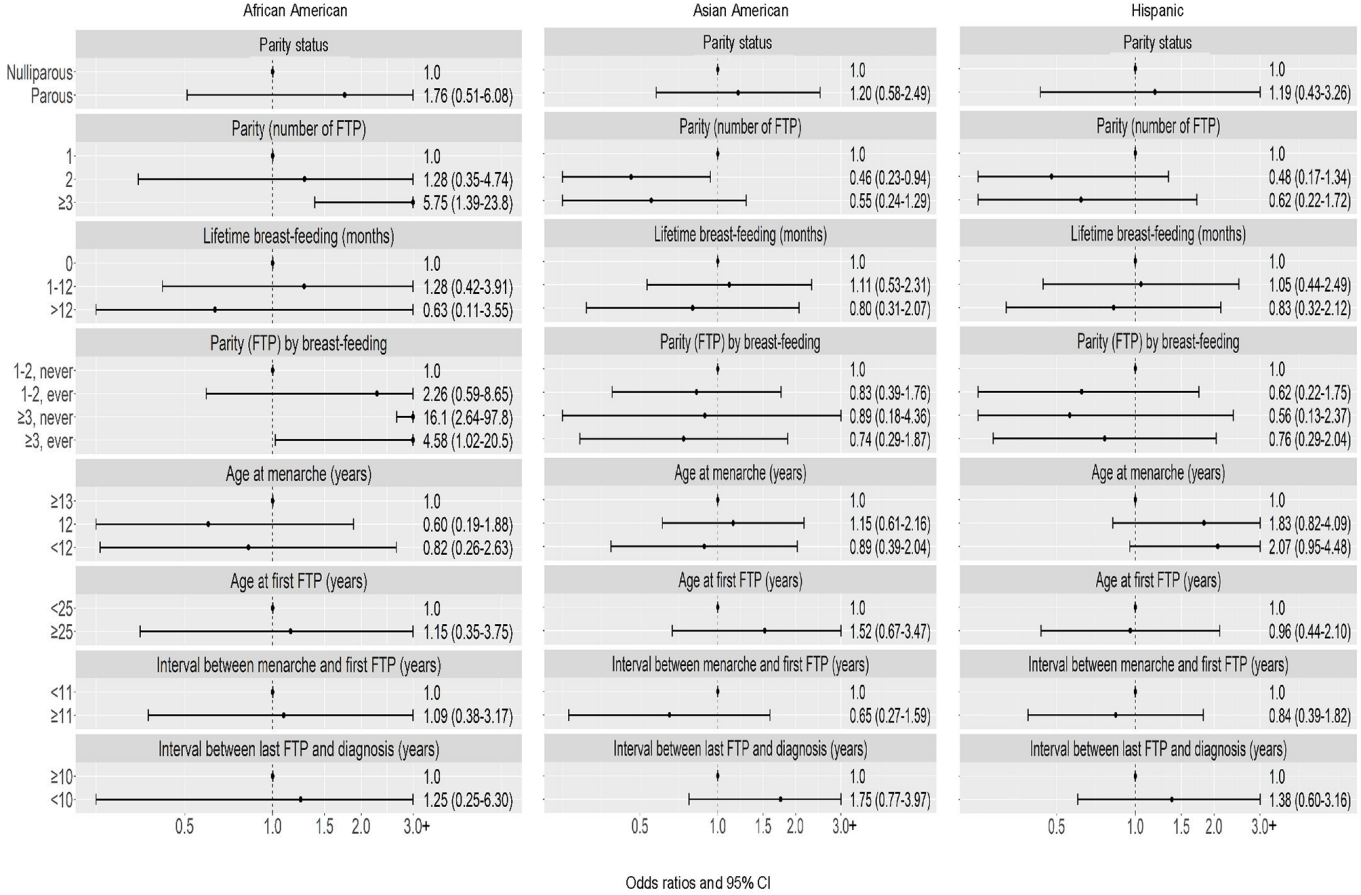
Triple-negative breast cancer: Associations with reproductive characteristics among premenopausal women, by race and ethnicity

No significant heterogeneity in associations by race and ethnicity was observed among premenopausal women (Table 6; Fig. 3); however, patterns of association were different with respect to TN subtype among premenopausal African American women. Higher parity was associated with higher risk of TN subtype (≥ 3 vs. 1 FTP: OR = 5.75, 95% CI = 1.39–23.8), and an even higher OR for the composite of higher parity without breast-feeding (OR = 16.1, 95% CI = 2.64–97.8). While the OR was attenuated for the composite of higher parity with breast-feeding, it remained elevated (OR = 4.58, 95% CI = 1.02–20.5).

**Table 6:** Triple-negative breast cancer: Associations with reproductive characteristics, by menopausal status and race and ethnicity

	All			African American			Asian American			Hispanic			Non-Hispanic White		
	Cs N	Cn N	OR (95% CI) ^a^	Cs N	Cn N	OR (95% CI) ^a^	Cs N	Cn N	OR (95% CI) ^a^	Cs N	Cn N	OR (95% CI) ^a^	Cs N	Cn N	OR (95% CI) ^a^
***Premenopausal women***	*264*	*1,929*		*50*	*195*		*64*	*1,036*		*79*	*523*		*71*	*175*	
***Parous premenopausal women***	*201*	*1,583*		*41*	*164*		*52*	*828*		*65*	*482*		*43*	*109*	
**Parity status**															
Nulliparous	63	346	1.0	9	31	1.0	12	208	1.0	14	41	1.0	28	66	1.0
Parous	201	1,583	1.27 (0.83–1.94)	41	164	1.76 (0.51–6.08)	52	828	1.20 (0.58–2.49)	65	482	1.19 (0.43–3.26)	43	109	1.39 (0.65–2.97)
p-heterogeneity ^b^ by race/ethnicity = 0.78															
p-heterogeneity ^c^ by menopausal status			0.03			0.39			0.40			0.56			0.03
**Parity (number of FTP)**															
1	58	340	1.0	9	48	1.0	22	212	1.0	12	52	1.0	15	28	1.0
2	71	655	0.52 (0.33–0.85)	13	65	1.28 (0.35–4.74)	18	401	0.46 (0.23–0.94)	22	131	0.48 (0.17–1.34)	18	58	0.41 (0.14–1.27)
≥ 3	72	588	0.92 (0.54–1.56)	19	51	5.75 (1.39–23.8)	12	215	0.55 (0.24–1.29)	31	299	0.62 (0.22–1.72)	10	23	0.94 (0.22–4.03)
p trend			0.79			0.01			0.11			0.60			0.68
p-heterogeneity ^b^ by race/ethnicity = 0.23															
p-heterogeneity ^c^ by menopausal status			0.05			0.04			0.66			0.35			0.07
**Lifetime breast-feeding (months), parous women**															
0	57	417	1.0	19	86	1.0	15	214	1.0	16	98	1.0	7	19	1.0
≤ 12	85	662	0.91 (0.57–1.46)	17	52	1.28 (0.42–3.91)	27	401	1.11 (0.53–2.31)	29	162	1.05 (0.44–2.49)	12	47	0.25 (0.06–1.11)
> 12	59	504	0.77 (0.45–1.32)	5	26	0.63 (0.11–3.55)	10	213	0.80 (0.31–2.07)	20	222	0.83 (0.32–2.12)	24	43	0.55 (0.13–2.33)
p trend			0.34			0.83			0.68			0.68			0.94
p-heterogeneity ^b^ by race and ethnicity = 0.37															
p-heterogeneity ^c^ by menopausal status			0.89			0.50			0.44			0.83			0.06
**Parity (FTP) by breast-feeding**															
1–2, never	41	308	1.0	9	63	1.0	14	181	1.0	12	50	1.0	6	14	1.0
1–2, ever	88	687	0.84 (0.50–1.41)	13	50	2.26 (0.59–8.65)	26	432	0.83 (0.39–1.76)	22	133	0.62 (0.22–1.75)	27	72	0.32 (0.07–1.42)
≥ 3, never	17	112	1.64 (0.73–3.68)	10	23	16.1 (2.64–97.8)	2	36	0.89 (0.18–4.36)	4	48	0.56 (0.13–2.37)	1	5	2.40 (0.12–47.5)
≥ 3, ever	55	476	1.07 (0.61–1.89)	9	28	4.58 (1.02–20.5)	10	179	0.74 (0.29–1.87)	27	251	0.76 (0.29–2.04)	9	18	0.63 (0.11–3.51)
p-heterogeneity ^b^ by race and ethnicity = 0.31															
p-heterogeneity ^c^ by menopausal status			0.20			0.01			0.74			0.87			0.15
**Age at menarche (years)**															
≥ 13	127	1,049	1.0	26	92	1.0	36	571	1.0	36	289	1.0	29	97	1.0
12	77	506	1.35 (0.92–1.99)	11	55	0.60 (0.19–1.88)	19	289	1.15 (0.61–2.16)	21	109	1.83 (0.82–4.09)	26	53	2.28 (1.00-5.16)
< 12	60	372	1.28 (0.84–1.97)	13	48	0.82 (0.26–2.63)	9	176	0.89 (0.39–2.04)	22	124	2.07 (0.95–4.48)	16	24	1.90 (0.76–4.74)
p trend			0.16			0.61			0.90			0.05			0.09
p-heterogeneity ^b^ by race/ethnicity = 0.19															
p-heterogeneity ^c^ by menopausal status			0.82			0.45			0.50			0.69			0.33
**Age at first FTP (years)**															
< 25	95	688	1.0	31	116	1.0	12	208	1.0	42	324	1.0	10	40	1.0
≥ 25	106	893	1.17 (0.74–1.85)	10	48	1.15 (0.35–3.75)	40	620	1.52 (0.67–3.47)	23	156	0.96 (0.44–2.10)	33	69	1.09 (0.31–3.82)
p-heterogeneity ^b^ by race and ethnicity = 0.92															
p-heterogeneity ^c^ by menopausal status			0.61			0.47			0.56			0.52			0.69
**Interval between menarche and first FTP (years)**															
< 11	80	586	1.0	26	102	1.0	10	164	1.0	37	288	1.0	7	32	1.0
≥ 11	121	993	1.37 (0.86–2.19)	15	62	1.09 (0.38–3.17)	42	664	0.65 (0.27–1.59)	28	191	0.84 (0.39–1.82)	36	76	0.62 (0.16–2.52)
p-heterogeneity ^b^ by race and ethnicity = 0.76															
p-heterogeneity ^c^ by menopausal status			0.94			0.98			0.34			0.64			0.95
**Interval between last FTP and diagnosis (years)**															
≥10	111	963	1.0	29	130	1.0	26	493	1.0	35	286	1.0	21	54	1.0
< 10	90	617	1.46 (0.89–2.39)	12	34	1.25 (0.25–6.30)	26	335	1.75 (0.77–3.97)	30	194	1.38 (0.60–3.16)	22	54	1.27 (0.34–4.77)
p-heterogeneity ^b^ by race and ethnicity = 0.98															
***Postmenopausal women*** ^***d***^	*293*	*2,438*		*60*	*430*		*66*	*904*		*75*	*867*		*92*	*237*	
***Parous postmenopausal women***	*234*	*2,177*		*50*	*381*		*54*	*775*		*71*	*823*		*59*	*198*	
**Parity status**															
Nulliparous	59	261	1.0	10	49	1.0	12	129	1.0	4	44	1.0	33	39	1.0
Parous	234	2,177	0.65 (0.43–0.99)	50	381	0.70 (0.24–2.09)	54	775	0.69 (0.31–1.50)	71	823	2.54 (0.62–10.4)	59	198	0.41 (0.20–0.83)
p-heterogeneity ^b^ by race and ethnicity = 0.08															
**Parity (number of FTP)**															
1	44	292	1.0	14	65	1.0	11	117	1.0	6	74	1.0	13	36	1.0
2	81	567	0.82 (0.50–1.35)	10	86	0.30 (0.08–1.08)	19	262	0.64 (0.27–1.54)	22	137	1.35 (0.41–4.46)	30	82	1.54 (0.60–3.95)
≥ 3	109	1,318	0.71 (0.43–1.18)	26	230	0.55 (0.17–1.78)	24	396	0.59 (0.24–1.43)	43	612	0.97 (0.30–3.12)	16	80	0.63 (0.20–1.95)
p trend			0.20			0.45			0.30			0.69			0.11
p-heterogeneity ^b^ by race and ethnicity = 0.49															
**Lifetime breast-feeding (months), parous women**															
0	102	763	1.0	36	200	1.0	25	225	1.0	26	254	1.0	15	84	1.0
≤ 12	76	742	0.84 (0.56–1.26)	11	104	0.77 (0.26–2.29)	20	317	0.57 (0.29–1.13)	19	249	0.75 (0.35–1.61)	26	72	1.53 (0.61–3.84)
> 12	56	672	0.84 (0.52–1.34)	3	77	0.21 (0.04–1.13)	9	233	0.58 (0.24–1.43)	26	320	0.95 (0.43–2.09)	18	42	1.57 (0.55–4.50)
p trend			0.41			0.08			0.13			0.86			0.39
p-heterogeneity ^b^ by race and ethnicity = 0.16															
**Parity (FTP) by breast-feeding**															
1–2, never	60	380	1.0	18	93	1.0	16	145	1.0	13	90	1.0	13	52	1.0
1–2, ever	65	479	0.85 (0.52–1.38)	6	58	0.52 (0.13–2.14)	14	234	0.49 (0.22–1.13)	15	121	0.84 (0.30–2.37)	30	66	1.11 (0.43–2.90)
≥ 3, never	42	385	0.87 (0.51–1.50)	18	107	1.03 (0.33–3.21)	9	82	0.75 (0.28-2.00)	13	164	0.82 (0.29–2.29)	2	32	0.23 (0.04–1.29)
≥ 3, ever	67	933	0.64 (0.40–1.03)	8	123	0.55 (0.16–1.92)	15	314	0.38 (0.17–0.84)	30	448	0.70 (0.29–1.67)	14	48	0.67 (0.22-2.00)
p-heterogeneity ^b^ by race and ethnicity = 0.39															
**Age at menarche (years)**															
≥ 13	162	1,402	1.0	32	241	1.0	41	526	1.0	43	504	1.0	46	131	1.0
12	63	547	1.13 (0.76–1.66)	8	104	0.87 (0.27–2.78)	16	222	1.06 (0.55–2.05)	16	168	2.14 (0.98–4.65)	23	53	1.05 (0.50–2.23)
< 12	66	479	0.98 (0.66–1.45)	20	84	1.63 (0.63–4.25)	7	156	0.39 (0.15–1.02)	16	187	1.28 (0.58–2.83)	23	52	1.35 (0.64–2.84)
p trend			0.99			0.37			0.10			0.37			0.45
p-heterogeneity ^b^ by race and ethnicity = 0.18															
**Age at first FTP (years)**															
< 25	141	1,273	1.0	41	321	1.0	22	297	1.0	49	544	1.0	29	111	1.0
≥ 25	93	893	0.97 (0.65–1.45)	9	60	2.11 (0.51–8.72)	32	478	0.82 (0.41–1.64)	22	268	0.75 (0.37–1.51)	30	87	1.51 (0.63–3.62)
p-heterogeneity ^b^ by race and ethnicity = 0.21															
**Interval between menarche and first FTP (years)**															
< 11	126	1,121	1.0	36	294	1.0	20	246	1.0	47	482	1.0	23	99	1.0
≥ 11	106	1,035	0.90 (0.61–1.34)	14	86	0.42 (0.12–1.44)	32	529	2.31 (1.11–4.81)	24	322	1.50 (0.74–3.04)	36	98	0.52 (0.22–1.23)
p-heterogeneity ^b^ by race and ethnicity = 0.01															

*AABCS* Asian American Breast Cancer Study, *BMI* body mass index, *FTP* full-term pregnancy, *NC-BCFR* Northern California Breast Cancer Family Registry, *SFBCS* San Francisco Bay Area Breast Cancer Study

^a^ Multivariable model was adjusted for study (AABCS, NC-BCFR, SFBCS); age (continuous) at diagnosis (cases) or selection/interview (controls); education (high school graduate or less, some college or vocational/technical school, college graduate or higher degree); family history of breast cancer in first-degree relatives (no, yes); personal history of benign breast disease (no, yes); parity (nulliparous, 1, 2, 3, ≥ 4 FTP); lifetime breast-feeding (nulliparous, 0, ≤ 12, >12 months); history of oral contraceptive use (never, former, current); composite variable of menopausal status and BMI (< 25, 25-29.9, ≥ 30); and alcohol consumption in reference year (0, < 6, ≥6 drinks/week)

^b^ P-heterogeneity by race and ethnicity using the Wald test

^c^ P-heterogeneity by menopausal status using the Wald test

^d^ Multivariable model for postmenopausal women was adjusted for covariates in footnote a, with history of oral contraceptive use categorized as ever vs. never use

**Fig. 4:**
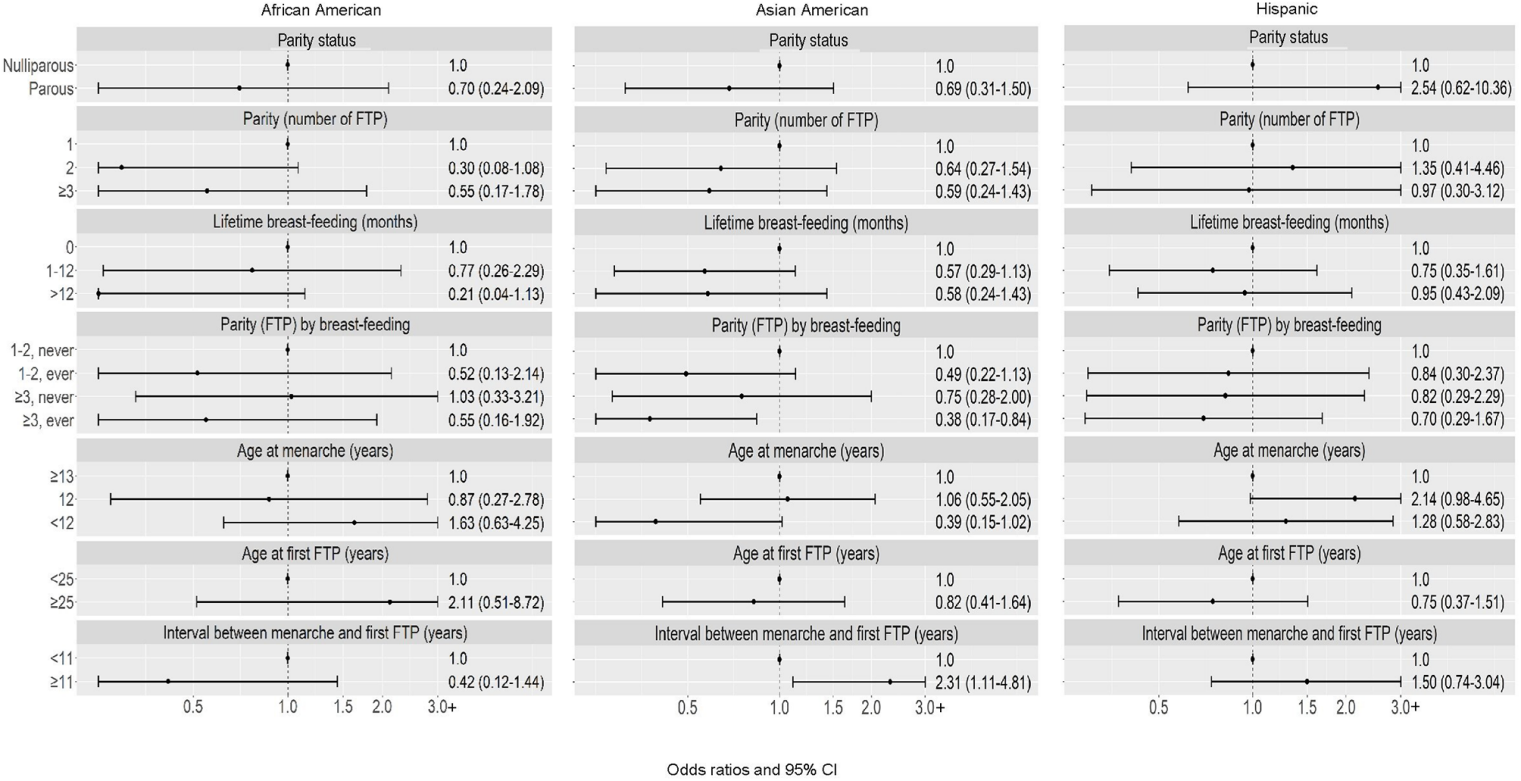
Triple-negative breast cancer: Associations with reproductive characteristics among postmenopausal women, by race and ethnicity

Among postmenopausal women, the composite of higher parity with breast-feeding was associated with lower risk of TN subtype, although the association was statistically significant among Asian American women only (OR = 0.38) (Fig. 4**)**. Heterogeneity by race and ethnicity was observed for the interval between menarche and first FTP (*p* = 0.01), with a higher risk associated with longer interval observed among Asian American women only (≥ 11 vs. <11 years: OR = 2.31).

#### *HER2-enriched subtype* (African American, Asian American, and Hispanic women)

Analyses of HER2-enriched subtype stratified by menopausal status and race and ethnicity were based on small sample sizes (Table 7). Among premenopausal Hispanic women, lower risk was associated with parity vs. nulliparity (OR = 0.19, p-heterogeneity by race and ethnicity < 0.01), and higher risk was associated with longer interval between menarche and first FTP (≥ 11 vs. <11 years: OR = 4.87). Among African American women, higher risk was associated with parity vs. nulliparity, higher parity, and a breast-feeding history, but OR estimates were based on very small case counts. Among postmenopausal women, higher parity was associated with lower risk among African American women (≥ 3 vs. 1–2 FTP: OR = 0.23), and younger age at menarche was associated with higher risk among Hispanic women (< 13 vs. ≥13 years: OR = 2.26).

**Table 7:** HER2-enriched breast cancer: Associations with reproductive characteristics, by menopausal status and race and ethnicity ^a^

	All			African American			Asian American			Hispanic		
	Cs N	Cn N	OR (95% CI) ^b^	Cs N	Cn N	OR (95% CI) ^b^	Cs	Cn	OR (95% CI) ^b^	Cs	Cn	OR (95% CI) ^b^
***Premenopausal women***	*107*	*1,754*		*16*	*195*		*49*	*1,036*		*42*	*523*	
***Parous premenopausal women***	*86*	*1,474*		*15*	*164*		*42*	*828*		*29*	*482*	
**Parity status**												
Nulliparous	21	280	1.0	1	31	1.0	7	208	1.0	13	41	1.0
Parous	86	1,474	0.86 (0.48–1.53)	15	164	1.96 (0.24–16.3)	42	828	1.56 (0.68–3.59)	29	482	0.19 (0.07–0.56)
p-heterogeneity ^c^ by race and ethnicity < 0.01												
p-heterogeneity ^d^ by menopausal status			0.95			0.37			0.27			0.09
**Parity (number of FTP)**												
1–2	61	909	1.0	11	113	1.0	34	613	1.0	16	183	1.0
≥ 3	25	565	0.89 (0.49–1.63)	4	51	5.25 (0.64–43.1)	8	215	0.61 (0.26–1.43)	13	299	1.08 (0.36–3.26)
p-heterogeneity ^c^ by race and ethnicity = 0.26												
p-heterogeneity ^d^ by menopausal status			0.20			0.04			0.95			0.58
**History of breast-feeding, parous women**												
Never	26	401	1.0	7	86	1.0	15	217	1.0	4	98	1.0
Ever	60	1,073	0.93 (0.53–1.64)	8	78	1.58 (0.36-7.00)	27	611	0.61 (0.30–1.21)	25	384	3.43 (0.78-15.0)
p-heterogeneity ^c^ by race and ethnicity = 0.24												
p-heterogeneity ^d^ by menopausal status			0.93			0.32			0.24			0.27
**Age at menarche (years)**												
≥ 13	57	952	1.0	11	92	1.0	24	571	1.0	22	289	1.0
< 13	49	801	1.18 (0.76–1.85)	5	103	0.23 (0.05–1.18)	24	465	1.26 (0.70–2.28)	20	233	1.22 (0.52–2.89)
p-heterogeneity ^c^ by race and ethnicity = 0.23												
p-heterogeneity ^d^ by menopausal status			0.47			0.03			0.27			0.13
**Age at first FTP (years)**												
< 25	34	648	1.0	9	116	1.0	8	208	1.0	17	324	1.0
≥ 25	52	824	1.80 (0.97–3.36)	6	48	1.31 (0.27–6.43)	34	620	1.32 (0.57–3.05)	12	156	3.70 (0.96–14.3)
p-heterogeneity ^c^ by race and ethnicity = 0.99												
p-heterogeneity ^d^ by menopausal status			0.57			0.37			0.90			0.17
**Interval between menarche and first FTP (years)**												
< 11	28	554	1.0	9	102	1.0	6	164	1.0	13	288	1.0
≥ 11	57	917	1.93 (1.01–3.68)	6	62	1.13 (0.24–5.43)	35	664	1.35 (0.54–3.40)	16	191	4.87 (1.30–18.2)
p-heterogeneity ^c^ by race and ethnicity = 0.86												
p-heterogeneity ^d^ by menopausal status			0.91			0.63			0.96			0.27
**Interval between last FTP and diagnosis (years)**												
≥ 10	46	909	1.0	9	102	1.0	6	164	1.0	13	288	1.0
< 10	40	563	1.64 (0.85–3.19)	5	34	1.87 (0.27–12.8)	19	335	1.49 (0.66–3.38)	16	194	1.87 (0.27–12.8)
p-heterogeneity ^c^ by race and ethnicity = 0.60												
***Postmenopausal women*** ^***e***^	*124*	*2,201*		*26*	*430*		*61*	*904*		*37*	*867*	
***Parous postmenopausal women***	*111*	*1,979*		*24*	*381*		*53*	*775*		*34*	*823*	
**Parity status**												
Nulliparous	13	222	1.0	2	49	1.0	8	129	1.0	3	44	1.0
Parous	111	1,979	1.12 (0.59–2.13)	24	381	1.81 (0.37–8.89)	53	775	0.89 (0.40–1.94)	34	823	1.02 (0.27–3.86)
p-heterogeneity ^c^ by race and ethnicity = 0.68												
**Parity (number of FTP)**												
1–2	65	741	1.0	18	151	1.0	33	379	1.0	14	211	1.0
≥ 3	46	1,238	0.70 (0.42–1.16)	6	230	0.23 (0.06–0.86)	20	396	0.64 (0.32–1.28)	20	612	1.36 (0.47–3.95)
p-heterogeneity ^c^ by race and ethnicity = 0.32												
**History of breast-feeding, parous women**												
Never	47	681	1.0	16	200	1.0	17	227	1.0	14	254	1.0
Ever	64	1,298	0.80 (0.51–1.25)	8	181	0.49 (0.15–1.62)	36	548	0.92 (0.49–1.75)	20	569	0.74 (0.35–1.60)
p-heterogeneity ^c^ by race and ethnicity = 0.79												
**Age at menarche (years)**												
≥ 13	63	1,271	1.0	10	241	1.0	37	526	1.0	16	504	1.0
< 13	59	921	1.30 (0.86–1.94)	16	188	2.54 (0.90–7.18)	23	378	0.77 (0.44–1.35)	20	355	2.26 (1.06–4.80)
p-heterogeneity ^c^ by race and ethnicity = 0.01												
**Age at first FTP (years)**												
< 25	62	1,162	1.0	20	321	1.0	17	297	1.0	25	544	1.0
≥ 25	49	806	0.85 (0.52–1.40)	4	60	0.36 (0.07–1.81)	36	478	1.25 (0.64–2.45)	9	268	0.64 (0.28–1.50)
p-heterogeneity ^c^ by race and ethnicity = 0.46												
**Interval between menarche and first FTP (years)**												
< 11	48	1,022	1.0	15	294	1.0	12	246	1.0	21	482	1.0
≥ 11	61	937	1.12 (0.67–1.87)	9	86	1.46 (0.44–4.84)	40	529	1.22 (0.58–2.57)	12	322	0.97 (0.43–2.20)
p-heterogeneity ^c^ by race and ethnicity = 0.66												

*AABCS* Asian American Breast Cancer Study, *BMI* body mass index, *FTP* full-term pregnancy, *NC-BCFR* Northern California Breast Cancer Family Registry, *SFBCS* San Francisco Bay Area Breast Cancer Study

^a^ Associations for NHW women were not assessed since the pooled dataset included only 10 NHW women with HER2-enriched breast cancer

^b^ Multivariable model was adjusted for study (AABCS, NC-BCFR, SFBCS); age (continuous) at diagnosis (cases) or selection/interview (controls); education (high school graduate or less, some college or vocational/technical school, college graduate or higher degree); family history of breast cancer in first-degree relatives (no, yes); personal history of benign breast disease (no, yes); parity (nulliparous, 1, 2, 3, ≥ 4 FTP); lifetime breast-feeding (nulliparous, 0, ≤ 12, >12 months); history of oral contraceptive use (never, former, current); and BMI (< 25, 25-29.9 ≥ 30); and alcohol consumption in reference year (0, < 6, ≥6 drinks/week)

^c^ P-heterogeneity by race and ethnicity using the Wald test

^d^ P-heterogeneity by menopausal status using the Wald test

^e^ Multivariable model for postmenopausal women was adjusted for covariates in footnote b, with history of oral contraceptive use categorized as ever vs. never use

## Discussion

To our knowledge, this is the only U.S. pooled study of breast cancer subtypes enriched with African American, Asian American, and Hispanic women. In the pooled dataset that comprised over 2,700 women with breast cancer, subtype-specific associations with reproductive factors were generally of similar magnitude across racial and ethnic groups and consistent with associations reported for NHW women. For luminal A subtype, lower risk associated with higher parity combined with a breast-feeding history was observed, regardless of menopausal status, with one exception. Among premenopausal African American women, higher parity without a breast-feeding history was associated with a higher risk of luminal A and TN subtypes; these higher risks, however, were attenuated by breast-feeding. For luminal A subtype among premenopausal women only, higher risk was associated with older age at first FTP, longer interval between menarche and first FTP, and shorter interval since last FTP, with similar OR estimates across the three racial and ethnic groups.

The two largest pooled analyses of breast cancer subtypes include an NCI Cohort Consortium analysis by Gaudet et al. (11,741 cases) [4] and an analysis of the Breast Cancer Association Consortium (BCAC) by Jung et al. (23,353 cases, 71,072 controls) [6]. Neither study presented racial- and ethnic-specific subtype results. Data are sparse for African American women on associations of reproductive factors with specific subtypes [21, 24, 25] or TN subtype [22, 23, 38]. The largest study for African American women to date is the African American Breast Cancer and Risk (AMBER) consortium (1,128 cases, 2,932 controls) [24]. To our knowledge, no prior studies have evaluated case-control associations with subtypes defined by joint ER/PR/HER2 status among Asian American and U.S. Hispanic women. Due to the diversity of the study sample (90% African American, Asian American, or Hispanic) and the over-sampling of TN cases in NC-BCFR, the proportions of women with luminal B (16%) and TN (21%) subtypes were higher in our study compared to U.S. population estimates [1].

For all women combined, the present findings of lower risk associated with parous status and higher parity (luminal A and luminal B) and longer breast-feeding (luminal A, HER2-enriched subtype, and TN of borderline statistical significance), and higher risk associated with older age at first FTP (luminal A subtype) were generally consistent with other studies [2, 4, 6, 7]. While some studies of breast cancer subtypes included only younger [12, 16] or older [13, 20] women, only a few studies stratified the analysis by menopausal status [17] or age [4, 6, 11, 21] for select reproductive factors. The present findings of heterogeneity by menopausal status for some reproductive variables highlight its importance, as associations could be masked without stratification. Among premenopausal African American women, we found no evidence of benefit associated with being parous or higher parity; in fact, higher ORs associated with higher parity were observed for all four subtypes, and the OR was statistically significant for TN subtype. For African American women overall, some studies found no evidence of higher risk of luminal A subtype associated with higher parity [21, 24], whereas other studies observed a higher risk of TN or basal-like subtypes [37, 38], likely reflecting the higher risk among premenopausal women only, since we found a strong inverse association with parity among postmenopausal African American women.

Although breast-feeding has been associated with lower risk of breast cancer, regardless of menopausal status [36], associations with breast cancer subtypes have not been consistent [3, 6, 40]. Some studies found similar risk reductions for luminal A and TN subtypes [21], or associations that were stronger for or limited to TN or basal-like subtypes [6, 12, 17, 24, 37]. Notably, in BCAC, a clear inverse association with breast-feeding was observed for TN subtype only [6]. In the present study, longer breast-feeding was associated with lower risk of luminal A, TN (borderline statistical significance), and HER2-enriched subtypes, although in analyses by race and ethnicity, none of the associations reached statistical significance. In agreement with a large pooled analysis of breast cancer overall [36], the risk reduction associated with higher parity was greater in the presence of a breast-feeding history among postmenopausal women for all four subtypes and among premenopausal women for luminal A and luminal B subtypes. Importantly, for luminal A, the most common subtype, this added benefit of breast-feeding was observed among all racial and ethnic and menopausal groups.

Our findings add to the growing evidence that breast-feeding may mitigate the higher risk of TN or ER-negative subtypes associated with higher parity [6, 18, 24, 37, 41]. It has been suggested that the mitigating effect of breast-feeding is more difficult to detect in populations with a high prevalence of breast-feeding [42]. We observed a mitigating effect among premenopausal African American women only who had the lowest prevalence of breast-feeding (48%) compared with 80% among premenopausal Hispanic control women. Pregnancy-associated breast cancer has been attributed to changes in pregnancy-related hormones, as well as immune factors and inflammatory processes triggered during postpartum involution that resemble the pro-tumorigenic process of wound healing. Specifically, the tissue microenvironment of involution, which includes the influx of immune cells, activated fibroblasts, extracellular matrix deposition, elevated matrix metalloproteinase levels, and bioactive matrix fragments, promotes tumorigenesis [43, 44].

We found that early menarche was associated with higher risk of luminal A subtype only and limited to premenopausal women, in agreement with two other pooled analyses that observed an association among younger women only [6, 21]. In contrast, early menarche was also associated with higher risk of non-luminal A subtypes, and in particular with TN subtype among younger women in BCAC [6]. Unlike some studies that observed a higher risk of luminal A subtype associated with earlier menarche among African American women [21, 24, 25], we found no association among African American women, although a longer interval between menarche and first FTP was associated with a suggestive higher risk of borderline statistical significance. The positive associations with luminal A subtype observed among Asian American and Hispanic women are consistent with other studies of NHW women [4, 17].

The exposure measure integrating two early reproductive events (age at menarche, age at first FTP) may be a more relevant risk factor for luminal A subtype, as this represents a window of increased susceptibility when breast tissue undergoes rapid cellular proliferation and rapid accumulation of risk until terminal differentiation occurs during a first pregnancy [45, 46]. The more than two-fold higher risk of premenopausal luminal A subtype associated with ≥ 15 vs. <10 years between menarche and first FTP is of particular concern given trends of delayed childbearing. We did not have data on exposures during this critical time window to further explore what factors might underlie this association, but additional research is warranted.

Pregnancy is associated with a transient increase in breast cancer risk that follows an FTP, wanes over time, and then shifts to a long-term reduction in breast cancer risk [47, 48], about 10 years after a last birth [6]. Consistent with these observations and the large BCAC analysis [6], a shorter interval (< 10 years) between last FTP and diagnosis was associated with a higher risk of luminal A subtype among premenopausal women. The overall OR estimate of 1.03 per year was the same across the three racial and ethnic groups, but reached statistical significance only for women overall.

### Comparisons across different subtype classifications

In analyses of mostly NHW women, associations with reproductive factors were generally of similar magnitude for subtypes defined by joint ER/PR/HER2 status or joint ER/PR status [4, 6, 18], and for ER-negative and TN subtypes [4, 6, 22]. Similarly, in our earlier BEM Study analysis [27], associations for ER/PR-positive breast cancer were similar to those for luminal A subtype in the present study, particularly for Asian American and Hispanic women. Larger studies will need to confirm the distinct associations we observed for luminal A vs. luminal B subtypes (e.g., breast-feeding among premenopausal women) and for TN vs. HER2-enriched subtypes (e.g., parity among postmenopausal women). In BCAC, associations with reproductive factors differed primarily between TN subtype and the other subtypes [6].

### Racial and ethnic differences in reproductive risk factors

Subtype-specific associations with reproductive factors among premenopausal and postmenopausal women were in the same direction and generally of similar magnitude across racial and ethnic groups, except for parity and breast-feeding among premenopausal African American women. Variation in OR estimates and very wide confidence intervals were likely due to small numbers, particularly among premenopausal women. Distributions of reproductive factors varied considerably across racial and ethnic groups which may contribute to racial and ethnic differences in the incidence of specific breast cancer subtypes. Palmer [22, 49] and Ambrosone [50] suggested that the higher prevalence of high parity, absence of breast-feeding, and young age at first FTP contributes to the higher incidence of early-onset ER-negative breast cancer among African American women. This constellation of factors may also contribute to the higher incidence of TN subtype among premenopausal African American women.

### Study limitations and strengths

The subtype-specific analyses were limited by sample size, especially for analyses of the less common subtypes stratified by menopausal status. Subtype was based on readily available cancer registry records, similar to other pooled analyses where subtype was based on medical records, pathology reports, or cancer registry data [4, 6]. The lack of centralized subtyping, as done in some studies [11, 12, 15, 17, 18, 24, 37], might have introduced some misclassification, but it is unlikely that such misclassification would be differential by reproductive characteristics. The small numbers of luminal A, luminal B, and HER2-enriched cases among NHW women precluded subtype-specific analyses in NHW women for comparison with published data from other studies. Not all eligible women with breast cancer and control women in the parent studies participated in the study interviews, which could have introduced selection bias. Reproductive characteristics were based on self-report, therefore subject to inaccurate recall. Non-differential recall bias could result in exposure misclassification which would bias the OR estimates towards the null. There is the possibility that recall is differential between cases and controls, although that may apply to a lesser extent for reproductive factors. Nevertheless, the associations for luminal A subtype in our study were generally consistent with the literature on breast cancer risk factors, providing support to the validity of our findings.

Study strengths include the population-based design of the three studies that were pooled, and case ascertainment through the regional population-based cancer registries which increases the generalizability of our study findings. The diversity of the study sample and use of harmonized exposure variables allowed the direct comparison of OR estimates for African American, Asian American, and Hispanic women. Detailed information was collected on pregnancy and breast-feeding histories and other risk factors. Lastly, we performed analyses stratified by menopausal status that revealed some important differences in associations.

### Implications for breast cancer prevention and risk reduction

Breast-feeding is likely the only reproductive risk factor for breast cancer that is potentially modifiable. Efforts focused on improving knowledge on the benefits of breast-feeding and creating a more supportive environment that facilitates breast-feeding could have major impact on lowering breast cancer risk for all subtypes, particularly among premenopausal African American women who are at higher risk. Breast-feeding disparities are tied at multiple levels to social determinants of health that impose barriers to breast-feeding, particularly among African American women (e.g., shorter parental leave; differential access to breast-feeding programs and lactation support; limited accommodations for pumping and storing breast milk at work; and historical and cultural factors [51–54]. Effective primary breast cancer prevention efforts focused on increasing breast-feeding need to address these barriers among African American women and implement tailored approaches that overcome them [54, 55]. The interval between menarche and first FTP may be a risk factor of increasing importance, given trends of earlier menarche [56, 57] and delayed childbearing [58]. Consistent with these trends, we saw a higher prevalence of longer mean interval between menarche and first FTP and a higher proportion of women with a first FTP at age ≥ 30 years among premenopausal compared to postmenopausal women. These findings warrant studies focused on identifying etiologic factors during this critical time window. The finding of a higher risk of luminal A subtype after a full-term pregnancy suggests that increased surveillance for breast cancer after a full-term pregnancy may be an important strategy to detect breast cancers at an early stage when they are easier to treat and have better survival.

## Conclusions

The higher incidence of TN and HER2-enriched breast cancer in some racial and ethnic groups [1], the worse prognosis for these subtypes [8], and the limited knowledge about risk factors warrant research focused on these less common subtypes. Foremost, larger studies and/or pooled analyses in racially and ethnically diverse populations are needed to evaluate reproductive and other risk factors for breast cancer subtypes with greater precision. The distinct associations with parity and breast-feeding among premenopausal African American women, as well as rising incidence rates of distant-stage breast cancer among women under age 40 years [59] underscore the importance of identifying risk factors for breast cancer subtypes among younger women. Centralized subtyping would minimize potential misclassification, and tumor expression data may further facilitate the detection of etiologic heterogeneity for more refined subtypes. A deeper understanding of subtype-specific risk factors, based on both menopausal status and race and ethnicity, is critical for prevention efforts aimed at reducing breast cancer risk and improving survival.

### Electronic supplementary material

Below is the link to the electronic supplementary material.

Supplementary Material 1

Supplementary Material 2

## Data Availability

The dataset used for the current study may be obtained from the corresponding author (EMJ) on reasonable request, contingent upon approval by appropriate Institutional Review Boards and study Principal Investigators.

## Abbreviations

AABCS: Los Angeles County Asian American Breast Cancer Study
BEM: Breast Cancer Etiology in Minorities
BMI: Body mass index
CI: Confidence interval
ER: Estrogen receptor
HER2: Human epidermal growth factor receptor 2
NC-BCFR: Northern California Breast Cancer Family Registry
OR: Odds ratio
PR: Progesterone receptor
SFBCS: San Francisco Bay Area Breast Cancer Study
U.S.: United States

## References

[1] Howlader N, Altekruse SF, Li CI, Chen VW, Clarke CA, Ries LA, Cronin KA. US incidence of breast cancer subtypes defined by joint hormone receptor and HER2 status. J Natl Cancer Inst. 2014;106(5). 10.1093/jnci/dju055 PMC4580552 24777111

[2] Barnard ME, Boeke CE, Tamimi RM (2015). Established breast cancer risk factors and risk of intrinsic tumor subtypes. Biochim Biophys Acta.

[3] Lambertini M, Santoro L, Del Mastro L, Nguyen B, Livraghi L, Ugolini D, Peccatori FA, Azim HA (2016). Jr. Reproductive behaviors and risk of developing breast cancer according to tumor subtype: a systematic review and meta-analysis of epidemiological studies. Cancer Treat Rev.

[4] Gaudet MM, Gierach GL, Carter BD, Luo J, Milne RL, Weiderpass E, Giles GG, Tamimi RM, Eliassen AH, Rosner B (2018). Pooled analysis of nine cohorts reveals breast cancer risk factors by tumor molecular subtype. Cancer Res.

[5] Houghton SC, Hankinson SE (2021). Cancer progress and priorities: breast cancer. Cancer Epidemiol Biomarkers Prev.

[6] Jung AY, Ahearn TU, Behrens S, Middha P, Bolla MK, Wang Q, Arndt V, Aronson KJ, Augustinsson A, Beane Freeman LE (2022). Distinct reproductive risk profiles for intrinsic-like breast cancer subtypes: pooled analysis of population-based studies. J Natl Cancer Inst.

[7] Mao X, Omeogu C, Karanth S, Joshi A, Meernik C, Wilson L, Clark A, Deveaux A, He C, Johnson T (2023). Association of reproductive risk factors and breast cancer molecular subtypes: a systematic review and meta-analysis. BMC Cancer.

[8] Howlader N, Cronin KA, Kurian AW, Andridge R (2018). Differences in breast cancer survival by molecular subtypes in the United States. Cancer Epidemiol Biomarkers Prev.

[9] Collaborative Group on Hormonal Factors in Breast Cancer (2012). Menarche, menopause, and breast cancer risk: individual participant meta-analysis, including 118 964 women with breast cancer from 117 epidemiological studies. Lancet Oncol.

[10] Kelsey JL, Gammon MD, John EM (1993). Reproductive factors and breast cancer. Epidemiol Rev.

[11] Ma H, Wang Y, Sullivan-Halley J, Weiss L, Marchbanks PA, Spirtas R, Ursin G, Burkman RT, Simon MS, Malone KE (2010). Use of four biomarkers to evaluate the risk of breast cancer subtypes in the women’s contraceptive and reproductive experiences study. Cancer Res.

[12] Gaudet MM, Press MF, Haile RW, Lynch CF, Glaser SL, Schildkraut J, Gammon MD, Douglas Thompson W, Bernstein JL (2011). Risk factors by molecular subtypes of breast cancer across a population-based study of women 56 years or younger. Breast Cancer Res Treat.

[13] Phipps AI, Chlebowski RT, Prentice R, McTiernan A, Wactawski-Wende J, Kuller LH, Adams-Campbell LL, Lane D, Stefanick ML, Vitolins M (2011). Reproductive history and oral contraceptive use in relation to risk of triple-negative breast cancer. J Natl Cancer Inst.

[14] Phipps AI, Buist DS, Malone KE, Barlow WE, Porter PL, Kerlikowske K, Li CI (2011). Reproductive history and risk of three breast cancer subtypes defined by three biomarkers. Cancer Causes Control.

[15] Tamimi RM, Colditz GA, Hazra A, Baer HJ, Hankinson SE, Rosner B, Marotti J, Connolly JL, Schnitt SJ, Collins LC (2012). Traditional breast cancer risk factors in relation to molecular subtypes of breast cancer. Breast Cancer Res Treat.

[16] Li CI, Beaber EF, Tang MT, Porter PL, Daling JR, Malone KE (2013). Reproductive factors and risk of estrogen receptor positive, triple-negative, and HER2-neu overexpressing breast cancer among women 20–44 years of age. Breast Cancer Res Treat.

[17] Sisti JS, Collins LC, Beck AH, Tamimi RM, Rosner BA, Eliassen AH (2016). Reproductive risk factors in relation to molecular subtypes of breast cancer: results from the nurses’ Health studies. Int J Cancer.

[18] Fortner RT, Sisti J, Chai B, Collins LC, Rosner B, Hankinson SE, Tamimi RM, Eliassen AH (2019). Parity, breastfeeding, and breast cancer risk by hormone receptor status and molecular phenotype: results from the nurses’ Health studies. Breast Cancer Res.

[19] McCarthy AM, Friebel-Klingner T, Ehsan S, He W, Welch M, Chen J, Kontos D, Domchek SM, Conant EF, Semine A (2021). Relationship of established risk factors with breast cancer subtypes. Cancer Med.

[20] Phipps AI, Malone KE, Porter PL, Daling JR, Li CI (2008). Reproductive and hormonal risk factors for postmenopausal luminal, HER-2-overexpressing, and triple-negative breast cancer. Cancer.

[21] Ma H, Ursin G, Xu X, Lee E, Togawa K, Duan L, Lu Y, Malone KE, Marchbanks PA, McDonald JA (2017). Reproductive factors and the risk of triple-negative breast cancer in white women and African-American women: a pooled analysis. Breast Cancer Res.

[22] Palmer JR, Viscidi E, Troester MA, Hong CC, Schedin P, Bethea TN, Bandera EV, Borges V, McKinnon C, Haiman CA et al. Parity, lactation, and breast cancer subtypes in African American women: results from the AMBER Consortium. J Natl Cancer Inst. 2014;106(10). 10.1093/jnci/dju237 PMC4271113 25224496

[23] Ambrosone CB, Zirpoli G, Hong CC, Yao S, Troester MA, Bandera EV, Schedin P, Bethea TN, Borges V, Park SY et al. Important role of menarche in development of estrogen receptor-negative breast cancer in African American women. J Natl Cancer Inst. 2015;107(9). 10.1093/jnci/djv172 PMC4836800 26085483

[24] Benefield HC, Zirpoli GR, Allott EH, Shan Y, Hurson AN, Omilian AR, Khoury T, Hong CC, Olshan AF, Bethea TN (2021). Epidemiology of basal-like and luminal breast cancers among black women in the AMBER Consortium. Cancer Epidemiol Biomarkers Prev.

[25] Friebel-Klingner TM, Ehsan S, Conant EF, Kontos D, Domchek SM, McCarthy AM (2021). Risk factors for breast cancer subtypes among black women undergoing screening mammography. Breast Cancer Res Treat.

[26] John EM, Hines LM, Phipps AI, Koo J, Longacre TA, Ingles SA, Baumgartner KB, Slattery ML, Wu AH (2018). Reproductive history, breast-feeding and risk of triple negative breast cancer: the breast Cancer etiology in minorities (BEM) study. Int J Cancer.

[27] John EM, Phipps AI, Hines LM, Koo J, Ingles SA, Baumgartner KB, Slattery ML, Wu AH (2020). Menstrual and reproductive characteristics and breast cancer risk by hormone receptor status and ethnicity: the breast Cancer etiology in minorities study. Int J Cancer.

[28] Clavel-Chapelon F (2002). Differential effects of reproductive factors on the risk of pre- and postmenopausal breast cancer. Results from a large cohort of French women. Br J Cancer.

[29] Trentham-Dietz A, Sprague BL, Hampton JM, Miglioretti DL, Nelson HD, Titus LJ, Egan KM, Remington PL, Newcomb PA (2014). Modification of breast cancer risk according to age and menopausal status: a combined analysis of five population-based case-control studies. Breast Cancer Res Treat.

[30] Chollet-Hinton L, Anders CK, Tse CK, Bell MB, Yang YC, Carey LA, Olshan AF, Troester MA (2016). Breast cancer biologic and etiologic heterogeneity by young age and menopausal status in the Carolina breast Cancer Study: a case-control study. Breast Cancer Res.

[31] Jeong SH, An YS, Choi JY, Park B, Kang D, Lee MH, Han W, Noh DY, Yoo KY, Park SK (2017). Risk reduction of breast cancer by childbirth, breastfeeding, and their interaction in Korean women: heterogeneous effects across menopausal status, hormone receptor status, and pathological subtypes. J Prev Med Public Health.

[32] Work ME, John EM, Andrulis IL, Knight JA, Liao Y, Mulligan AM, Southey MC, Giles GG, Dite GS, Apicella C (2014). Reproductive risk factors and oestrogen/progesterone receptor-negative breast cancer in the breast Cancer Family Registry. Br J Cancer.

[33] Wu AH, Vigen C, Lee E, Tseng CC, Butler LM (2016). Traditional breast cancer risk factors in Filipina americans compared with Chinese and Japanese americans in Los Angeles County. Cancer Epidemiol Biomarkers Prev.

[34] John EM, Phipps AI, Davis A, Koo J (2005). Migration history, acculturation, and breast cancer risk in hispanic women. Cancer Epidemiol Biomarkers Prev.

[35] John EM, Sangaramoorthy M, Koo J, Whittemore AS, West DW (2019). Enrollment and biospecimen collection in a multiethnic family cohort: the Northern California site of the breast Cancer Family Registry. Cancer Causes Control.

[36] Collaborative Group on Hormonal Factors in Breast Cancer (2002). Breast cancer and breastfeeding: collaborative reanalysis of individual data from 47 epidemiological studies in 30 countries, including 50302 women with breast cancer and 96973 women without the disease. Lancet.

[37] Millikan RC, Newman B, Tse CK, Moorman PG, Conway K, Smith LV, Labbok MH, Geradts J, Bensen JT, Jackson S (2008). Epidemiology of basal-like breast cancer. Breast Cancer Res Treat.

[38] Ambrosone CB, Zirpoli G, Ruszczyk M, Shankar J, Hong CC, McIlwain D, Roberts M, Yao S, McCann SE, Ciupak G (2014). Parity and breastfeeding among African-American women: differential effects on breast cancer risk by estrogen receptor status in the women’s Circle of Health Study. Cancer Causes Control.

[39] Amadou A, Hainaut P, Romieu I (2013). Role of obesity in the risk of breast cancer: lessons from anthropometry. J Oncol.

[40] Islami F, Liu Y, Jemal A, Zhou J, Weiderpass E, Colditz G, Boffetta P, Weiss M (2015). Breastfeeding and breast cancer risk by receptor status–a systematic review and meta-analysis. Ann Oncol.

[41] Ambrosone CB, Higgins MJ (2020). Relationships between breast feeding and breast cancer subtypes: lessons learned from studies in humans and in mice. Cancer Res.

[42] Schedin P, Palmer JR (2022). Can breast Cancer Prevention Strategies be tailored to Biologic Subtype and Unique Reproductive Windows?. J Natl Cancer Inst.

[43] Schedin P (2006). Pregnancy-associated breast cancer and metastasis. Nat Rev Cancer.

[44] Lyons TR, Schedin PJ, Borges VF (2009). Pregnancy and breast cancer: when they collide. J Mammary Gland Biol Neoplasia.

[45] Colditz GA, Bohlke K, Berkey CS (2014). Breast cancer risk accumulation starts early: prevention must also. Breast Cancer Res Treat.

[46] Colditz GA, Frazier AL (1995). Models of breast cancer show that risk is set by events of early life: prevention efforts must shift focus. Cancer Epidemiol Biomarkers Prev.

[47] Lambe M, Hsieh C, Trichopoulos D, Ekbom A, Pavia M, Adami HO (1994). Transient increase in the risk of breast cancer after giving birth. N Engl J Med.

[48] Albrektsen G, Heuch I, Kvale G (1995). The short-term and long-term effect of a pregnancy on breast cancer risk: a prospective study of 802,457 parous Norwegian women. Br J Cancer.

[49] Palmer JR, Boggs DA, Wise LA, Ambrosone CB, Adams-Campbell LL, Rosenberg L (2011). Parity and lactation in relation to estrogen receptor negative breast cancer in African American women. Cancer Epidemiol Biomarkers Prev.

[50] Ambrosone CB, Zirpoli GR, Bovbjerg DH, Shankar J, Hong CC, McCann SE, Ruszczyk M, Khoury T, Yao S, Ciupak GL (2014). Associations between estrogen receptor-negative breast cancer and timing of reproductive events differ between African American and European American women. Cancer Epidemiol Biomarkers Prev.

[51] Jones KM, Power ML, Queenan JT, Schulkin J (2015). Racial and ethnic disparities in breastfeeding. Breastfeed Med.

[52] Li R, Perrine CG, Anstey EH, Chen J, MacGowan CA, Elam-Evans LD (2019). Breastfeeding trends by race/ethnicity among US children born from 2009 to 2015. JAMA Pediatr.

[53] Morrow AL, McClain J, Conrey SC, Niu L, Kinzer A, Cline AR, Piasecki AM, DeFranco E, Ward L, Ware J (2021). Breastfeeding disparities and their mediators in an urban birth cohort of Black and White mothers. Breastfeed Med.

[54] Segura-Perez S, Hromi-Fiedler A, Adnew M, Nyhan K, Perez-Escamilla R (2021). Impact of breastfeeding interventions among United States minority women on breastfeeding outcomes: a systematic review. Int J Equity Health.

[55] Johnson A, Kirk R, Rosenblum KL, Muzik M (2015). Enhancing breastfeeding rates among African American women: a systematic review of current psychosocial interventions. Breastfeed Med.

[56] Wyshak G, Frisch RE (1982). Evidence for a secular trend in age of menarche. N Engl J Med.

[57] Morris DH, Jones ME, Schoemaker MJ, Ashworth A, Swerdlow AJ (2011). Secular trends in age at menarche in women in the UK born 1908-93: results from the breakthrough generations study. Paediatr Perinat Epidemiol.

[58] Kehm RD, Osypuk TL, Poynter JN, Vock DM, Spector LG. Do pregnancy characteristics contribute to rising childhood cancer incidence rates in the United States? Pediatr Blood Cancer. 2018;65(3). 10.1002/pbc.26888 PMC5766387 29160610

[59] Kehm RD, Yang W, Tehranifar P, Terry MB (2019). 40 years of change in age- and stage-specific cancer incidence rates in US women and men. JNCI Cancer Spectr.

