# Supplementary material for: Reproductive characteristics, menopausal status, race and ethnicity, and risk of breast cancer subtypes defined by ER, PR and HER2 status: the Breast Cancer Etiology in Minorities study
Source: Breast Cancer Res. 2024 May 31;26:88. doi: 10.1186/s13058-024-01834-5 (PMC11143591; doi:10.1186/s13058-024-01834-5)
Supplement: Supplementary file 1 — Supplementary Material 1 [file 13058_2024_1834_MOESM1_ESM.docx]

**Supplemental Table S1** Description of studies included in the pooled analysis

| **Study** | **Age at diagnosis**  **Cancer Registry Geographic area** | **Case Selection** | **Case Sampling Criteria** | **Control Selection** | **Cases with first primary invasive breast cancer and controls** |
| --- | --- | --- | --- | --- | --- |
| AABCS ^a^ | 25-74 years  Los Angeles County Cancer Surveillance Program  Los Angeles county | First primary breast cancer  1995-2001  2003-2006  Chinese, Filipina, Japanese | All Chinese, Filipina, Japanese cases | Neighborhood controls (block-walking)  1:1 case:control ratio  Frequency matched to cases on Asian ethnicity and +/- 5 years | 1,818 cases  (746 Chinese, 644 Filipina, 428 Japanese)  1,911 controls  (869 Chinese, 550 Filipina, 492 Japanese) |
| SFBCS ^b^ | 35-79 years  Greater Bay Area Cancer Registry  San Francisco Bay Area (5 counties) | First primary invasive breast cancer  1995-1999: African American, non-Hispanic White  1995-2002: Hispanic | All African American and Hispanic cases, 10% random sample of non-Hispanic White cases | Random digit dialing  1:1 case-control ratio  Frequency matched to cases on race and ethnicity and 5-year age group  1:1.5 case-control ratio for Hispanics (1995-1998) | 2,256 cases  (543 African American, 1,118 Hispanic, 595 non-Hispanic White)    2,706 controls  (598 African American, 1,462 Hispanic, 646 non-Hispanic White) |

| NC-BCFR ^c^ | 18-64 years  Greater Bay Area Cancer Registry  San Francisco Bay Area (8 counties)  Sacramento and Sierra Cancer Registries  Sacramento region (2 counties, dx 2005-2006) | First or second primary breast cancer  1995-1998: all race/ethnicities  1999-2002: Chinese, Japanese, Filipina, African American, Hispanic  2003-2009: African American, Hispanic  2007-2009: all triple negative cases  2005-2006: African American, Hispanic | All cases with indicators of increased genetic susceptibility (diagnosis at age <35 years, personal or first-degree family history of breast, ovarian or childhood cancer)  Random samples of cases diagnosed at age 35-64 years: 2.5% non-Hispanic Whites, 33% others (African American, Chinese, Filipina, Japanese, Hispanic) | Random digit dialing  2:1 case:control ratio  Frequency matched to cases diagnosed from 1995-1998 on race and ethnicity and 5-year age group | 2,778 cases  (611 African American, 729 Asian American, 776 Hispanic, 645 non-Hispanic White, 17 mixed ethnicity)    626 controls  (87 Asian American, 73 Hispanic, 73 African American, 387 non-Hispanic White, 6 mixed ethnicity) |
| --- | --- | --- | --- | --- | --- |

*AABCS* Asian American Breast Cancer Study, *dx* diagnosis, *NC-BCFR* Northern California Breast Cancer Family Registry, *SFBCS* San Francisco Bay Area Breast Cancer Study

**References**

^a^ Wu AH, Vigen C, Lee E, Tseng CC, Butler LM. Traditional Breast Cancer Risk Factors in Filipina Americans Compared with Chinese and Japanese Americans in Los Angeles County. Cancer Epidemiol Biomarkers Prev. 2016;25(12):1572-1586.

^b^ John EM, Phipps AI, Davis A, Koo J. Migration history, acculturation, and breast cancer risk in Hispanic women. Cancer Epidemiol Biomarkers Prev. 2005;14(12):2905-2913.

^c^ John EM, Sangaramoorthy M, Koo J, Whittemore AS, West DW. Enrollment and biospecimen collection in a multiethnic family cohort: the Northern California site of the Breast Cancer Family Registry. Cancer Causes Control. 2019;30(4):395-40

**Supplemental Table S2** Analytic sample of controls and breast cancer cases by subtype, race and ethnicity, menopausal status, and parity status

|  | All | African American | Asian American | Hispanic | Non-Hispanic  White |
| --- | --- | --- | --- | --- | --- |
| ***Premenopausal women*** | | | | | |
| Controls | 1929 | 195 | 1036 | 523 | 175 |
| Cases | 1291 | 198 | 544 | 426 | 113 |
| Luminal A | 699 | 94 | 327 | 246 | 32 ^a^ |
| Luminal B | 215 | 38 | 104 | 69 | 4 ^a^ |
| Triple negative | 264 | 50 | 64 | 79 | 71 |
| HER2-enriched | 113 | 16 | 49 | 42 | 6 ^a^ |
| ***Parous premenopausal women*** | | | | | |
| Controls | 1583 | 164 | 828 | 482 | 109 |
| Cases | 962 | 160 | 388 | 345 | 69 |
| Luminal A | 511 | 76 | 223 | 192 | 20 ^a^ |
| Luminal B | 160 | 28 | 71 | 59 | 2 ^a^ |
| Triple negative | 201 | 41 | 52 | 65 | 43 |
| HER2-enriched | 90 | 15 | 42 | 29 | 4 ^a^ |
| ***Postmenopausal women*** | | | | | |
| Controls | 2438 | 430 | 904 | 867 | 237 |
| Cases | 1428 | 268 | 540 | 468 | 152 |
| Luminal A | 792 | 150 | 313 | 281 | 48 ^a^ |
| Luminal B | 216 | 32 | 100 | 75 | 9 ^a^ |
| Triple negative | 293 | 60 | 66 | 75 | 92 |
| HER2-enriched | 127 | 26 | 61 | 37 | 3 ^a^ |
| ***Parous postmenopausal women*** | | | | | |
| Controls | 2177 | 381 | 775 | 823 | 198 |
| Cases | 1182 | 215 | 433 | 425 | 109 |
| Luminal A | 659 | 116 | 246 | 257 | 40 ^a^ |
| Luminal B | 175 | 25 | 80 | 63 | 7 ^a^ |
| Triple negative | 234 | 50 | 54 | 71 | 59 |
| HER2-enriched | 114 | 24 | 53 | 34 | 3 ^a^ |
|  |  |  |  |  |  |

^a^ NHW cases were not included in the analyses of luminal A, luminal B, and HER2-enriched subtypes by menopausal status and race and ethnicity.

**Table S3** Reproductive characteristics of controls, by menopausal status and race and ethnicity

|  |  |  |  |  |  |  |  |  |
| --- | --- | --- | --- | --- | --- | --- | --- | --- |
|  | African American | | Asian American | | Hispanic | | NHW | |
|  |  |  |  |  |  |  |  |  |
|  | N | % | N | % | N | % | N | % |
|  |  |  |  |  |  |  |  |  |
| ***Premenopausal women*** ^a^ |  |  |  |  |  |  |  |  |
|  |  |  |  |  |  |  |  |  |
| Parity (number of FTP) |  |  |  |  |  |  |  |  |
| Nullipaorus | 31 | 16 | 208 | 20 | 41 | 8 | 66 | 38 |
| 1 | 48 | 25 | 212 | 20 | 52 | 10 | 28 | 16 |
| 2 | 65 | 33 | 401 | 39 | 131 | 25 | 58 | 33 |
| 3 | 25 | 13 | 157 | 15 | 143 | 27 | 12 | 7 |
| ≥4 | 26 | 13 | 58 | 6 | 156 | 30 | 11 | 6 |
|  |  |  |  |  |  |  |  |  |
| Lifetime breast-feeding (months), parous women |  |  |  |  |  |  |  |  |
| 0 | 86 | 52 | 214 | 26 | 98 | 20 | 19 | 17 |
| ≤12 | 52 | 32 | 401 | 48 | 162 | 34 | 47 | 43 |
| >12 - 24 | 16 | 10 | 132 | 16 | 98 | 20 | 28 | 26 |
| >24 | 10 | 6 | 81 | 10 | 124 | 26 | 15 | 14 |
| Mean duration of breast-feeding (months) | 7.0 |  | 7.3 |  | 18.5 |  | 9.2 |  |
|  |  |  |  |  |  |  |  |  |
| Age at menarche (years) |  |  |  |  |  |  |  |  |
| <12 | 42 | 22 | 291 | 28 | 161 | 31 | 39 | 22 |
| 12 | 50 | 26 | 280 | 27 | 128 | 25 | 58 | 33 |
| 13 | 55 | 28 | 289 | 28 | 109 | 21 | 53 | 30 |
| ≥14 | 48 | 25 | 176 | 17 | 124 | 24 | 24 | 14 |
| Mean age (years) | 12.5 |  | 12.8 |  | 12.8 |  | 12.7 |  |
|  |  |  |  |  |  |  |  |  |
| Age at first FTP (years) |  |  |  |  |  |  |  |  |
| <20 | 56 | 34 | 37 | 4 | 159 | 33 | 5 | 5 |
| 20-24 | 60 | 37 | 171 | 21 | 165 | 34 | 35 | 32 |
| 25-29 | 29 | 18 | 314 | 38 | 90 | 19 | 33 | 30 |
| ≥30 | 19 | 12 | 306 | 37 | 66 | 14 | 36 | 33 |
| Mean age at FTP (years) | 22.3 |  | 28.1 |  | 22.9 |  | 27.3 |  |
|  |  |  |  |  |  |  |  |  |
| Interval between menarche and first FTP (years) |  |  |  |  |  |  |  |  |
| <10 | 93 | 57 | 121 | 15 | 256 | 53 | 26 | 24 |
| 10-14 | 38 | 23 | 254 | 31 | 122 | 25 | 31 | 29 |
| ≥15 | 33 | 20 | 453 | 55 | 101 | 21 | 51 | 47 |
| Mean interval (years) | 9.9 |  | 15.2 |  | 10.1 |  | 14.7 |  |
|  |  |  |  |  |  |  |  |  |
| Interval between last FTP and diagnosis (years) |  |  |  |  |  |  |  |  |
| ≥20 | 61 | 37 | 105 | 13 | 73 | 15 | 19 | 18 |
| 10-19 | 69 | 42 | 388 | 47 | 213 | 44 | 35 | 32 |
| <10 | 34 | 21 | 335 | 40 | 194 | 40 | 54 | 50 |
|  |  |  |  |  |  |  |  |  |
| ***Postmenopausal women*** ^a^ |  |  |  |  |  |  |  |  |
|  |  |  |  |  |  |  |  |  |
| Parity (number of FTP) |  |  |  |  |  |  |  |  |
| Nullipaorus | 49 | 11 | 129 | 14 | 44 | 5 | 39 | 16 |
| 1 | 65 | 15 | 117 | 13 | 74 | 9 | 36 | 15 |
| 2 | 86 | 20 | 262 | 29 | 137 | 16 | 82 | 35 |
| 3 | 97 | 23 | 194 | 21 | 187 | 22 | 42 | 18 |
| ≥4 | 133 | 31 | 202 | 22 | 425 | 49 | 38 | 16 |
|  |  |  |  |  |  |  |  |  |
| Lifetime breast-feeding (months), parous women |  |  |  |  |  |  |  |  |
| 0 | 200 | 52 | 225 | 29 | 254 | 31 | 84 | 42 |
| ≤12 | 104 | 27 | 317 | 41 | 249 | 30 | 72 | 36 |
| >12 - 24 | 36 | 9 | 119 | 15 | 118 | 14 | 22 | 11 |
| >24 | 41 | 11 | 114 | 15 | 202 | 25 | 20 | 10 |
| Mean duration of breast-feeding (months) | 8.6 |  | 10.5 |  | 21.2 |  | 7.6 |  |
|  |  |  |  |  |  |  |  |  |
| Age at menarche (years) |  |  |  |  |  |  |  |  |
| <12 | 123 | 29 | 318 | 35 | 306 | 36 | 53 | 22 |
| 12 | 118 | 28 | 208 | 23 | 198 | 23 | 78 | 33 |
| 13 | 104 | 24 | 222 | 25 | 168 | 20 | 53 | 22 |
| ≥14 | 84 | 20 | 156 | 17 | 187 | 22 | 52 | 22 |
| Mean age (years) | 12.9 |  | 13.0 |  | 12.9 |  | 12.6 |  |
|  |  |  |  |  |  |  |  |  |
| Age at first FTP (years) |  |  |  |  |  |  |  |  |
| <20 | 182 | 48 | 49 | 6 | 226 | 28 | 32 | 16 |
| 20-24 | 139 | 36 | 248 | 32 | 318 | 39 | 79 | 40 |
| 25-29 | 40 | 10 | 325 | 42 | 187 | 23 | 56 | 28 |
| ≥30 | 20 | 5 | 153 | 20 | 81 | 10 | 31 | 16 |
| Mean age at FTP (years) | 20.8 |  | 25.9 |  | 22.9 |  | 24.0 |  |
|  |  |  |  |  |  |  |  |  |
| Interval between menarche and first FTP (years) |  |  |  |  |  |  |  |  |
| <10 | 278 | 73 | 198 | 26 | 425 | 53 | 82 | 42 |
| 10-14 | 69 | 18 | 312 | 40 | 238 | 30 | 65 | 33 |
| ≥15 | 33 | 9 | 265 | 34 | 141 | 18 | 50 | 25 |
| Mean interval (years) | 7.9 |  | 12.9 |  | 10.0 |  | 11.4 |  |
|  |  |  |  |  |  |  |  |  |

*FTP* full-term pregnancy

^a^ Chi-square p value <0.05 for difference by race and ethnicity for all reproductive characteristics shown in the table
