# Supplementary material for: Reproductive characteristics, menopausal status, race and ethnicity, and risk of breast cancer subtypes defined by ER, PR and HER2 status: the Breast Cancer Etiology in Minorities study
Source: Breast Cancer Res. 2024 May 31;26:88. doi: 10.1186/s13058-024-01834-5 (PMC11143591; doi:10.1186/s13058-024-01834-5)

Additional file 4. Figure S1. Luminal A breast cancer: Associations with reproductive characteristics among all women combined and by menopausal status


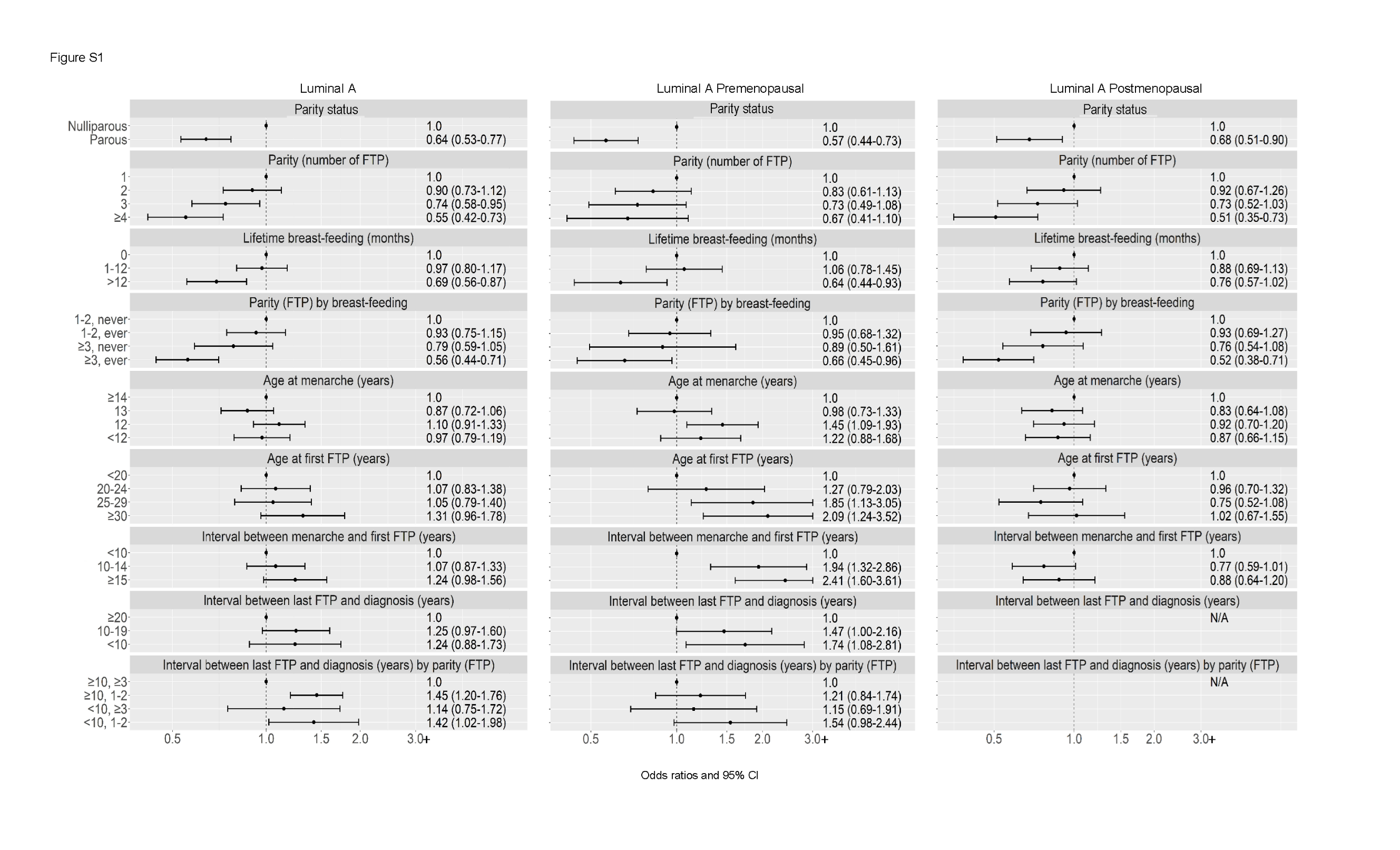


Additional file 5. Figure S2. Luminal B breast cancer: Associations with reproductive characteristics among all women combined and by menopausal status


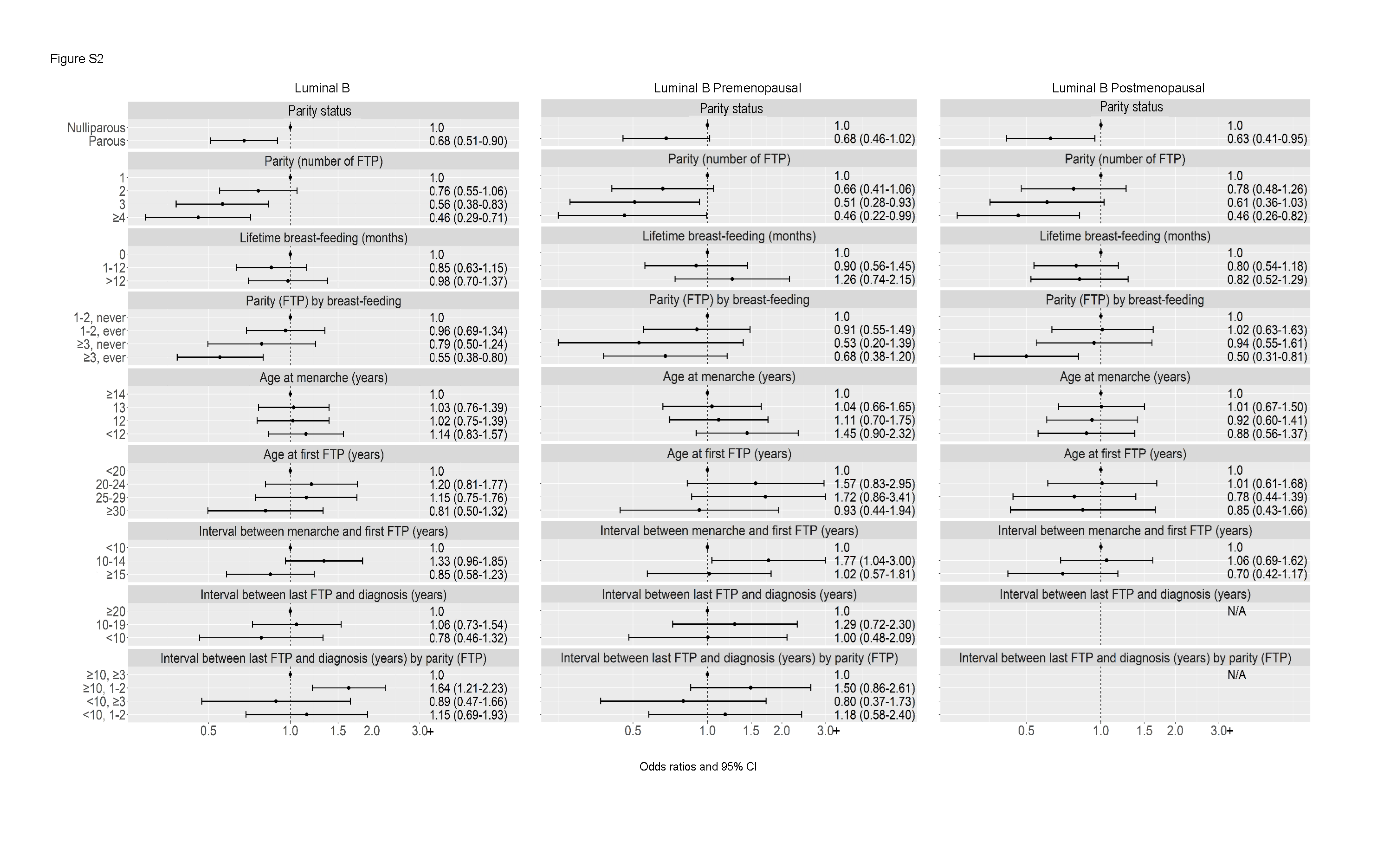


Additional file 6. Figure S3. Triple-negative breast cancer: Associations with reproductive characteristics among all women combined and by menopausal status


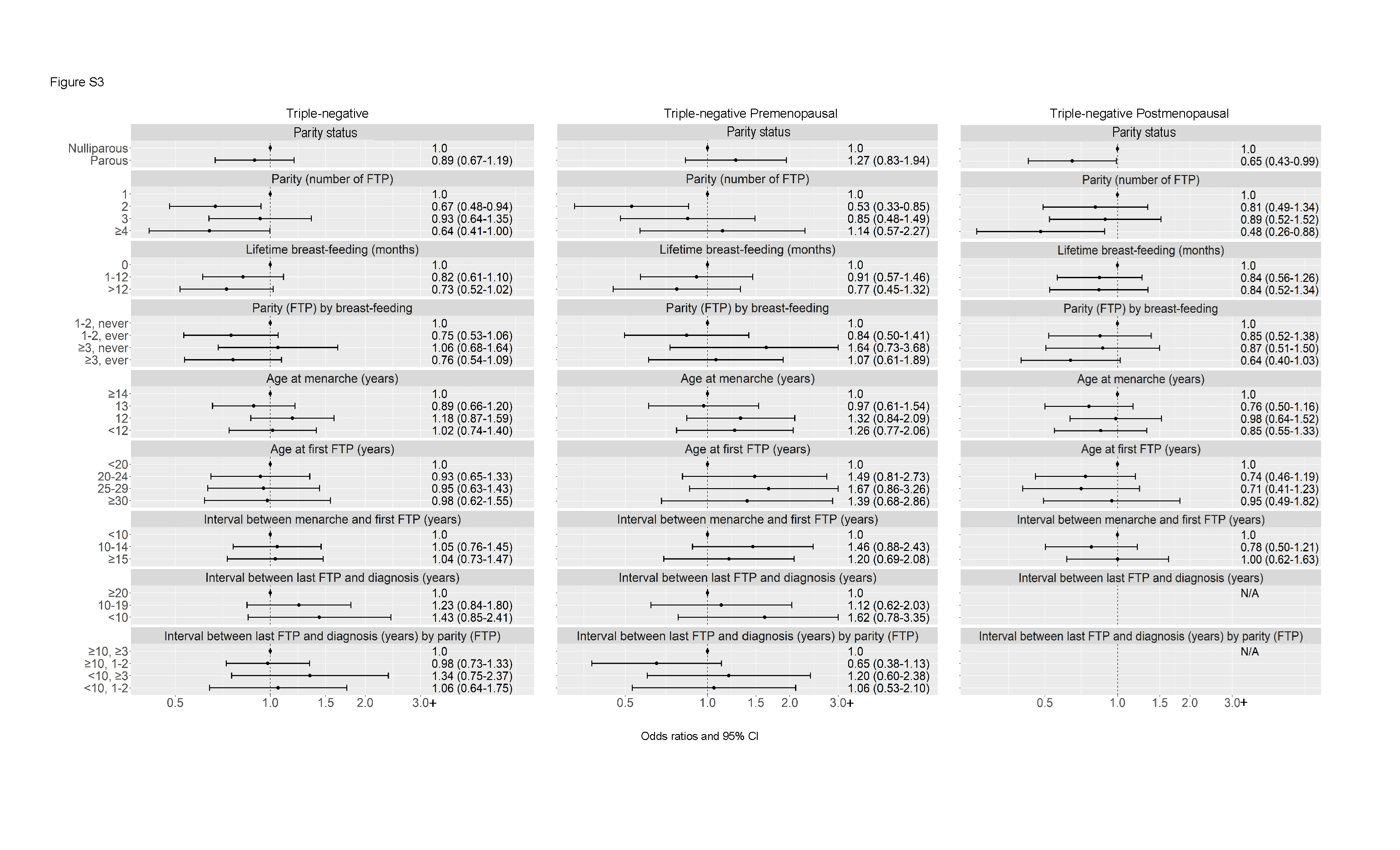


Additional file 7. Figure S4. HER2-enriched breast cancer: Associations with reproductive characteristics among all women combined and by menopausal status
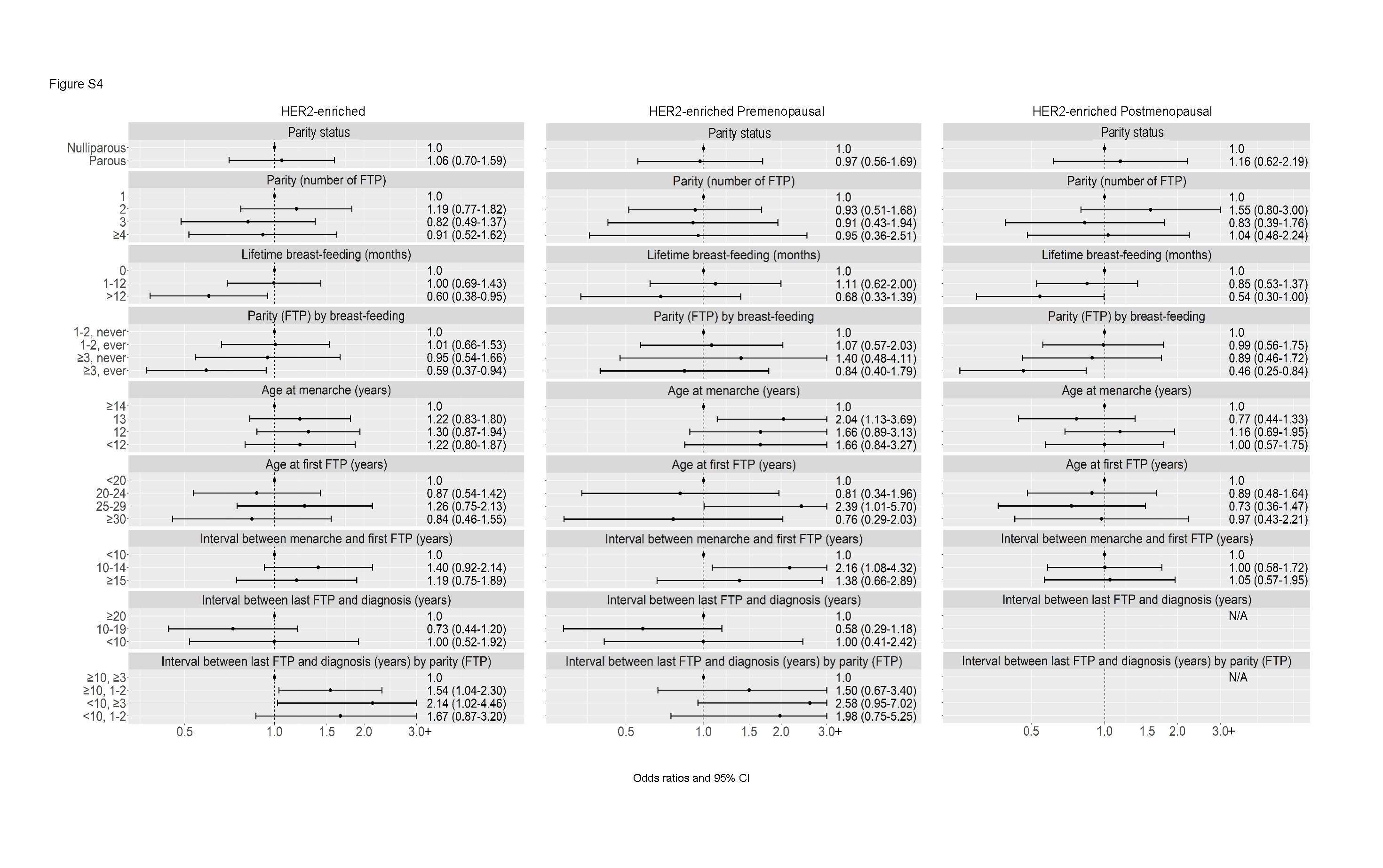

Supplement: Supplementary file 2 — Supplementary Material 2 [file 13058_2024_1834_MOESM2_ESM.docx]
